# The Tomato Brown Rugose Fruit Virus Movement Protein Gene Is a Novel Microbial Source Tracking Marker

**DOI:** 10.1128/aem.00583-23

**Published:** 2023-07-05

**Authors:** Aravind Natarajan, Brayon J. Fremin, Danica T. Schmidtke, Marlene K. Wolfe, Soumaya Zlitni, Katherine E. Graham, Erin F. Brooks, Christopher J. Severyn, Kathleen M. Sakamoto, Norman J. Lacayo, Scott Kuersten, Jeff Koble, Glorianna Caves, Inna Kaplan, Upinder Singh, Prasanna Jagannathan, Andrew R. Rezvani, Ami S. Bhatt, Alexandria B. Boehm

**Affiliations:** a Department of Genetics, Stanford University, Stanford, California, USA; b Department of Medicine (Hematology, Blood and Marrow Transplantation), Stanford University, Stanford, California, USA; c Department of Microbiology and Immunology, Stanford University, Stanford, California, USA; d Department of Civil and Environmental Engineering, Stanford University, Stanford, California, USA; e Department of Pediatrics, (Hematology/Oncology/Stem Cell Transplant & Regenerative Medicine), Stanford University, Stanford, California, USA; f Illumina, Inc., San Diego, California, USA; g Department of Medicine (Blood and Marrow Transplantation and Cellular Therapy), Stanford University, Stanford, California, USA; h Department of Medicine (Infectious Diseases and Geographic Medicine), Stanford University, Stanford, California, USA; University of Nebraska—Lincoln

**Keywords:** tomato brown rugose fruit virus, human-associated marker, MST, virus, ToBRFV, wastewater, fecal pollution, microbial source tracking

## Abstract

Microbial source tracking (MST) identifies sources of fecal contamination in the environment using host-associated fecal markers. While there are numerous bacterial MST markers that can be used herein, there are few such viral markers. Here, we designed and tested novel viral MST markers based on tomato brown rugose fruit virus (ToBRFV) genomes. We assembled eight nearly complete genomes of ToBRFV from wastewater and stool samples from the San Francisco Bay Area in the United States. Next, we developed two novel probe-based reverse transcription-PCR (RT-PCR) assays based on conserved regions of the ToBRFV genome and tested the markers’ sensitivities and specificities using human and non-human animal stool as well as wastewater. The ToBRFV markers are sensitive and specific; in human stool and wastewater, they are more prevalent and abundant than a commonly used viral marker, the pepper mild mottle virus (PMMoV) coat protein (CP) gene. We used the assays to detect fecal contamination in urban stormwater samples and found that the ToBRFV markers matched cross-assembly phage (crAssphage), an established viral MST marker, in prevalence across samples. Taken together, these results indicate that ToBRFV is a promising viral human-associated MST marker.

**IMPORTANCE** Human exposure to fecal contamination in the environment can cause transmission of infectious diseases. Microbial source tracking (MST) can identify sources of fecal contamination so that contamination can be remediated and human exposures can be reduced. MST requires the use of host-associated MST markers. Here, we designed and tested novel MST markers from genomes of tomato brown rugose fruit virus (ToBRFV). The markers are sensitive and specific to human stool and highly abundant in human stool and wastewater samples.

## INTRODUCTION

Across the world, water quality is assessed for human fecal contamination using microbial indicators, including enterococci and total coliforms like Escherichia coli (1–3). Using these organisms to assess water quality is advantageous because they are abundant in human stool, which enables detection of even trace contamination of water bodies. Additionally, their presence may indicate the potential contamination of water bodies by other, sparser human pathogens that may be harder to detect. However, there are limitations to their utility. These microbial indicators of human fecal contamination are also found in non-human stool (4). Additionally, they can be present and even grow in the environment, including in decaying plant material (1, 5) and in soils and sands (6, 7). Therefore, there is a need to identify new microbial indicator targets that can be used to specifically assess the presence of human fecal contamination.

The process of detecting microbes and identifying sources of microbial contamination in the environment is known as microbial source tracking (MST). MST targets have also been used in severe acute respiratory syndrome coronavirus 2 (SARS-CoV-2) wastewater-based epidemiology applications as “fecal strength” and endogenous extraction controls (8). Over the last decade, sensitive and specific molecular MST markers have been developed for various animal stools, including those from humans (9), cows (10), and birds (11). Most of these MST markers target conserved regions of bacterial genomes (9), with the exception of two that target viruses, the cross-assembly phage (crAssphage) (12) and pepper mild mottle virus (PMMoV) (13). crAssphage, a phage of *Bacteroidetes*, is a DNA virus that is highly abundant in human stool (14). PMMoV is a plant RNA virus found at high concentrations in human stool given its presence in popular spices, hot sauces, and other food products (15). The performance of MST targets is evaluated in terms of sensitivity and specificity for a given host’s stool. For instance, a sensitive target for human stool is present at high concentrations in nearly all human fecal samples, so that dilute human stool can be detected in the environment. Meanwhile, a specific target is absent in nearly all non-human fecal samples. A previous study defined an MST assay as being sensitive and specific if the true positive and true negative rates were greater than 80% (9).

In this study, we present a new human-associated, RNA-based, viral MST target that is highly abundant in human stool and wastewater, tomato brown rugose fruit virus (ToBRFV). ToBRFV was first identified in Israel in 2014 and has since been detected across the world. As of early 2023, ToBRFV had been found across four continents, in at least 35 countries; this is likely an underestimate (16). We assembled eight nearly complete genomes of ToBRFV from wastewater and stool samples from the San Francisco Bay Area (Bay Area) in California in the United States, representing some of the first complete genomes from stool and wastewater in the area. Using these complete genomes and other publicly available genomes, we developed two novel hydrolysis probe-based reverse transcription-PCR (RT-PCR) assays based on conserved regions of its RNA genome and tested their sensitivity and specificity using stool and wastewater samples. Finally, we used this assay for MST in stormwater samples collected from an urban environment. With the finding that ToBRFV is a reliable RNA-virus based MST marker, this study makes a valuable contribution to detecting human fecal contamination of the environment and to wastewater-based epidemiology.

## RESULTS AND DISCUSSION

### ToBRFV is widely prevalent and abundant in sequence data from stool and wastewater samples.

Tracking the presence of human feces in the environment and identifying internal controls for the processing of stool and wastewater samples require marker genes that are (i) prevalent, i.e., consistently present across samples, and (ii) abundant, i.e., at high enough concentration for reliable detection. crAssphage (12) is one such DNA-based marker, and PMMoV is an RNA-based marker (13). We sought to identify the most abundant and prevalent source of RNA from RNA sequencing data from human stool and wastewater samples.

**FIG 1:**
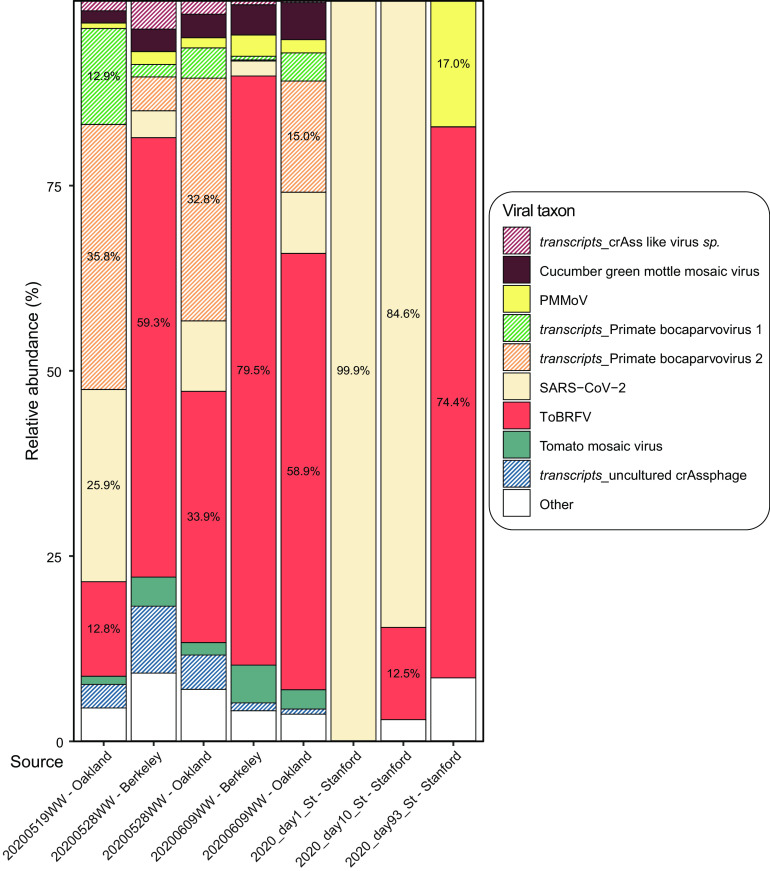
Relative abundance of viral RNA from sequencing wastewater and stool samples. The *x* axis represents the source of the eight sequencing data sets analyzed here. Five wastewater samples are marked by the date of collection in YYYYMMDD format followed by “WW” and the location of collection. The three stool samples are marked by the year and timepoint of collection followed by “St” and the location of collection. The *y* axis indicates the relative abundance of each taxon. The color scheme represents specific taxa as shown in the color key. Patterned bars highlight sequence reads from transcripts from taxa that have DNA genomes. For taxa with >10.0% relative abundance, the percent abundance is also presented in the histogram.

We isolated and sequenced RNA from three longitudinal stool samples from one human participant who had tested positive for SARS-CoV-2. In parallel, we acquired publicly available transcriptomics data from five wastewater samples that had been collected and sequenced from the Bay Area (17). Using these sequence data from eight samples, we identified all represented RNA viruses and their relative abundances (Fig. 1). ToBRFV was the most widely prevalent RNA virus, present in all five wastewater samples and three stool samples. It was detected at very low relative abundance (0.077% of viral reads) in one of the stool samples during the time of active SARS-CoV-2 infection, in which 99.9% of viral reads belonged to SARS-CoV-2. In the other seven samples where it was detected, it was the only viral RNA with a relative abundance consistently over 10.0% in viral reads, often making up over 50.0% of the reads. Notably, the relative abundance of ToBRFV was consistently greater than that of PMMoV, which is a well-established MST marker and known to be highly abundant in wastewater (8). This is consistent with reports from studies carried out prior to (17) and in parallel (18, 19) with ours that also show that ToBRFV is a highly prevalent virus in wastewater.

### Novel ToBRFV genomes and sequence analysis reveal suitable RNA-borne marker genes.

Having determined that ToBRFV is a prevalent and abundant RNA virus in sequence data, we next set out to identify genomic regions suitable as targets for primers/probes for its reliable molecular detection.

**FIG 2:**
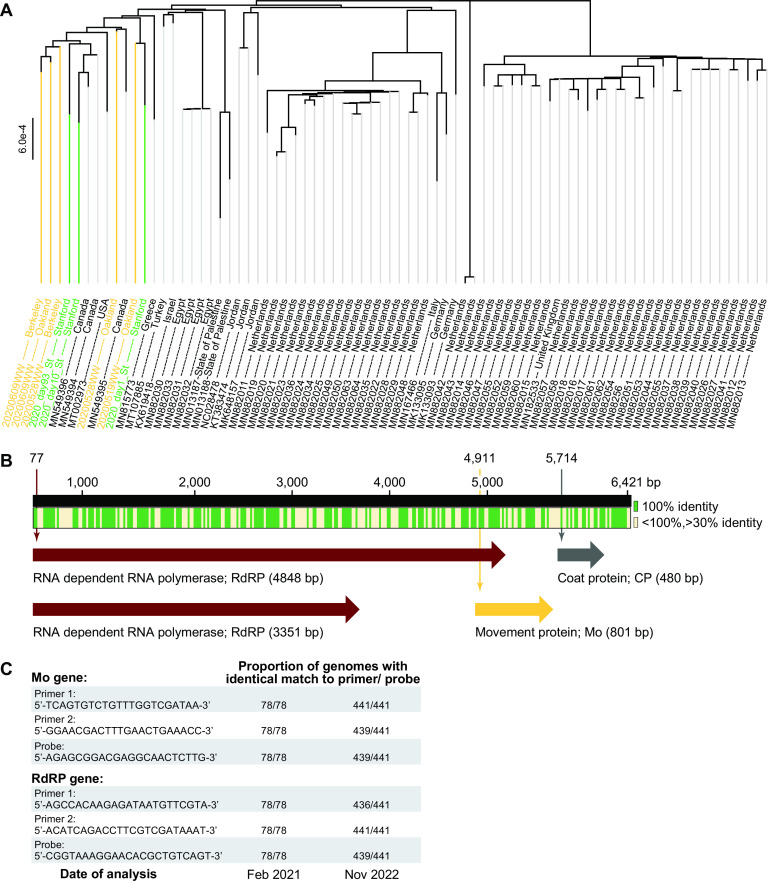
Analysis of newly assembled ToBRFV genomes and generation of primer/probe sets for ddRT-PCR. (A) Phylogenetic tree of 78 nearly complete genomes of ToBRFV, including eight genomes generated in the current study from wastewater and stool. All genomes are listed by their NCBI accession number and source location. Seventy preexisting genomes are listed in black font, five genomes derived from wastewater samples are in yellow, and three from stool samples are in green. (B) Summary of multiple-sequence alignment and gene annotation across the 78 ToBRFV genomes. Green indicates regions that are 100.0% conserved across all genomes, while cream marks those that are greater than 30.0% but less than 100.0% conserved. Two variants of the RdRP-encoding gene are found at 77 bp and are either 4,848 bp or 3,351 bp in size. The Mo protein-encoding gene is found at 4,911 bp and is 801 bp in size. The CP-encoding gene is found at 5,714 bp and is 480 bp in size. Genomic locations are based on the genome ID NC_028478. (C) Sequences of primer/probe sets generated in the current study, in February 2021, aimed at targeting the Mo and RdRP genes across all known genomes. Since the number of known genomes grew from February 2021 to November 2022, the final column indicates the proportion of the 441 current genomes bearing sequences identical to the designed primer/probe sets.

In February 2021, at the start of this study, only 70 nearly complete ToBRFV genomes were known. Fifty of these were from the Netherlands. None had been sequenced from human stool or wastewater samples, and only one sequence was from the United States. In order to ensure that the assay we developed was universal, we first decided to augment the number of ToBRFV genomes and the diversity of their sources. Therefore, we assembled nearly complete genomes of ToBRFV using sequence data generated in this study from stool samples and using existing data from wastewater samples (17), both collected in the Bay Area. The eight newly assembled genomes had a mean completeness of 98.8% (range, 93.6% to 100.0%; median, 99.4%) (see Table S5 in the supplemental material). The longitudinally acquired stool samples yielded ToBRFV genomes with single nucleotide polymorphisms (SNPs) in 27 positions, suggesting possible strain variation over time. Looking more broadly, across all 78 nearly complete ToBRFV genomes, we identified 2,808 positions containing SNPs (across an average contig length of 6,366 bp), and the 12 North American strains form their own distinct cluster (Fig. 2B).

Multiple-sequence analysis across all 78 ToBRFV genomes highlights regions that are 100.0% conserved (Fig. 2C). Among these, gene annotation reveals (i) two variants of the RNA-dependent RNA polymerase (RdRP)-encoding gene at 2,700 bp on the chromosome, which differ by whether an internal stop codon is read through (size, 3,351 bp or 4,848 bp), (ii) the movement protein (Mo)-encoding gene (size, 480 bp) at 5,166 bp, and (iii) the coat protein (CP)-encoding gene (size, 801 bp) at 5,166 bp (Fig. 2C). Among these, we designed primer/probe sets targeting the 5′ end of the RdRP gene and the Mo gene. We were unable to identify a suitable primer set for the CP gene for droplet digital RT-PCR (ddRT-PCR). Notably, the primer/probe sets designed here (Table 1) were conserved across all 78 genomes (Fig. 2D).

**TABLE 1:** Primers and probes designed in this study to quantify ToBRFV Mo and RdRP genes

Primer or probe	Description	Sequence (5′ to 3′)	Amplicon length (bp) or modifications^*a*^
Primers			
ToBRFV_Mo_F	ToBRFV Mo gene; forward primer	TCA GTG TCT GTT TGG TCG ATA A	105
ToBRFV_Mo_R	ToBRFV Mo gene; reverse primer	GGA ACG ACT TTG AAC TGA AAC C	
ToBRFV_RdRP_F	ToBRFV RdRP gene; forward primer	AGC CAC AAG AGA TAA TGT TCG TA	103
ToBRFV_RdRP_R	ToBRFV RdRP gene; reverse primer	ACA TCA GAC CTT CGT CGA TAA AT	
Probes			
ToBRFV_Mo_P	ToBRFV Mo gene; probe	AGA GCG GAC GAG GCA ACT CTT G	FAM/ZEN/IBHQ
ToBRFV_RdRP_P	ToBRFV RdRP gene; probe	ACG GTA AAG GAA CAC GCT GTC AGT	FAM/ZEN/IBHQ

a FAM, 6-carboxyfluorescein; ZEN, proprietary to IDT; IBHQ, 3’-Iowa Black Fluorescent Quencher.

Between the first phase of this study in February 2021 and the completion of this work in November 2022, the number of nearly complete ToBRFV genomes increased to 441 (Table 2), with additional genomes from Belgium, France, Mexico, Switzerland, and the United States. Therefore, we repeated the phylogenetic analysis of the novel genomes generated in the current study in the context of all 441 currently known genomes (Fig. S3). Again, we found that the genomes derived from North America cluster distinctly. Finally, we analyzed whether the primer/probe sets proposed here continue to be universal and found that the oligonucleotides targeting Mo are a perfect sequence match in 439/441 genomes, while those targeting RdRP are a perfect match in 436/441 genomes (Fig. 2C).

**TABLE 2:** Sources of genomes analyzed

Sample type	Sample source	No. of ToBRFV genomes available in:		Reference or source	
		Feb 2021	Nov 2022	Sequence data	Assembled genomes
Stool	Bay Area, CA, USA	3	0	This study	This study
Tomatoes	Global	70	183	NA	https://www.ncbi.nlm.nih.gov/nuccore
Wastewater	Southern CA, USA	0	250	NA	18
Wastewater	Bay Area, CA, USA	5	0	NCBI BioProject (PRJNA661613)	This study

### ToBRFV-targeting primer/probe sets have low limit of blank and limit of detection.

Having newly designed primer/probe sets targeting the Mo and RdRP genes in ToBRFV, we aimed to validate these oligonucleotides and establish the limits of their reliable utility.

To this end, we acquired synthetic DNA constructs featuring regions of the ToBRFV Mo and RdRP genes targeted by hydrolysis-probe RT-PCR assays from Integrated DNA Technologies (IDT) cloned into the pIDT plasmid. We also acquired a similar plasmid containing the PMMoV CP gene. Using ddRT-PCR, we assayed a dilution series of these synthetic plasmid constructs at 1, 2, 5, 10, 100, and 1,000 copies/μL of template in triplicate and found that all the primer/probe sets detected the target gene at all concentrations (Fig. S4). Next, we focused our attention on the negative controls included in the assays to identify the limit of detection (LoD) for each primer/probe set. The negative controls included two no-template controls, water and RNAlater, and two mismatched controls that were the synthetic pIDT plasmids bearing targets orthogonal to the primer/probe sets. Therefore, theoretically, all the negative controls would have no detectable gene target. For each primer/probe set, among the negative controls, we identified the highest concentration of target detected and set this value as the limit of blank (LoB). This means that any concentration below −0.552 log_10_ copies/μL of template for the primer/probe set targeting PMMoV CP gene, −0.590 log_10_ copies/μL of template for the ToBRFV Mo gene, and 0.407 log_10_ copies/μL of template for the ToBRFV RdRP gene is not reliable (Fig. S4). After converting all concentrations of gene targets below the LoB to zero, we focused our attention on the triplicate dilution series to identify the lowest concentration of template at which all three reactions had a detectable target concentration (Fig. S4). We set this concentration as the LoD, i.e., the lowest concentration at which a gene target can be reliably detected. The LoD for the primer/probe set targeting the PMMoV CP gene was 1 copy/μL of template, that for the ToBRFV Mo gene was 5 copies/μL of template, and that for the ToBRFV RdRP gene was 5 copies/μL of template. All gene target concentrations below the LoD were set to zero.

### ToBRFV was not detected in stool from non-human animals.

**FIG 3:**
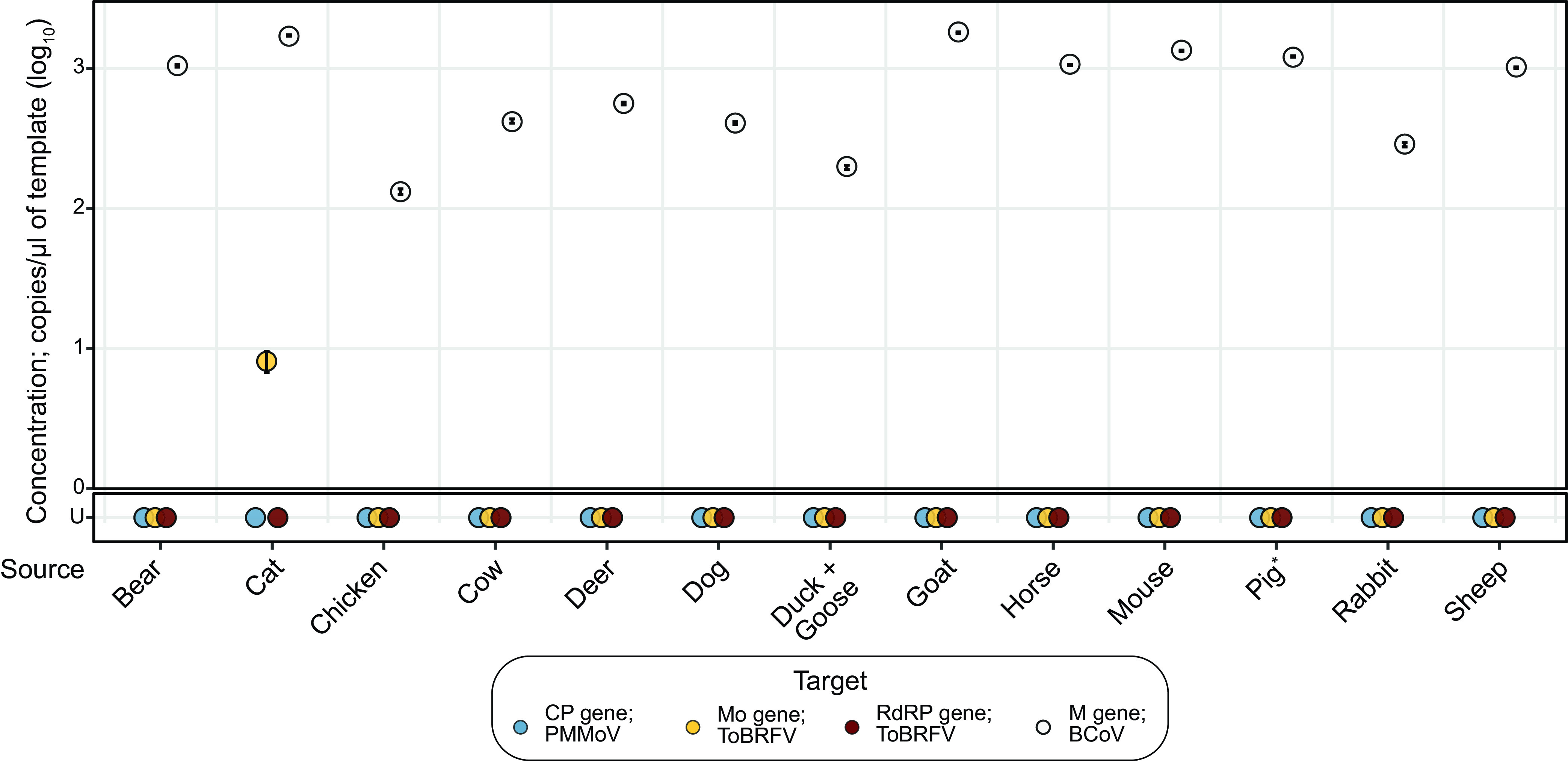
Concentrations of PMMoV and ToBRFV target genes in animal stool samples. Dot plot marking the concentrations of the PMMoV CP (blue), ToBRFV Mo (yellow) and RdRP (red) genes from the undiluted templates. Concentrations of BCoV M gene, used as a control, are marked by white dots. Error bars marking the standard deviation are plotted along with the dots and are mostly subsumed within the dots. The *x* axis shows 13 samples from 14 different animals, as a single sample was derived from cohoused ducks and geese. The *y* axis shows concentrations of the genes. U, undetermined (samples with no detectable gene). *Note that 1:10-diluted RNA template from a pig’s stool indicated 7.47 log_10_ copies/μL of template of the PMMoV CP gene.

MST targets should be specific, meaning that they are mostly absent in stool from other common animals. Therefore, having established that our primer/probe sets are functional, we tested them against stool collected from 14 different animals, including wild bear and deer, chickens, cows, ducks, geese, goats, and sheep from a farm, horses and pigs from a barn, a household cat, dog, and rabbit, and laboratory mice. Notably, these animals are rather diverse and are fed a wide variety of foods. While RNA extracted from all of these animal samples had a detectable concentration of the M gene target from the spiked-in bovine coronavirus (BCoV) used as a control, none of them had RNA containing either the PMMoV CP gene or the ToBRFV RdRP gene (Fig. 3). The ToBRFV Mo gene was detectable only in the sample derived from the domesticated cat, perhaps due to inclusion of tomatoes in its processed kibble or cross contamination of its diet with that of its human cohabitant. Therefore, all three primer/probe sets to detect RNA from PMMoV and ToBRFV do not detect RNA in most animal feces, except for the ToBRFV Mo gene in a cat, indicating that they are specific for human stool.

To test whether the absence of PMMoV and ToBRFV gene targets in these samples was an artifact of inhibited RT-PCR, we diluted the RNA extracts 1:10 and assayed for the gene targets from PMMoV and ToBRFV. Results obtained with the diluted template were the same as those obtained with undiluted template, indicating the absence of inhibition, with one exception. In the assay for the PMMoV CP gene, we found that the diluted template from pig’s stool yielded a detectable concentration of 7.47 log_10_ copies/μL of template, suggesting that this animal may have ingested some PMMoV as part of its diet and that the corresponding assay with the undiluted template was affected by PCR inhibition.

### Description of participants who provided human stool samples used for RNA quantification.

Analyzing sequence information from three stool samples collected from one human participant revealed ToBRFV to be abundantly present. To further test the sensitivity of the assays to human stool, we relied on a stool biobank including 194 stool samples from 125 adults and 28 samples from four children, all of whom were undergoing hematopoietic cell transplantation (HCT), cell therapy (CAR-T [chimeric antigen receptor T cell]), or induction chemotherapy for the treatment of underlying hematologic disorders.

Of the adult participants, 79 were male, 45 were female, and 1 did not provide information on their sex. The median age of the adult participants was 60 years (range, 19 to 82 years), and that of the pediatric participants was 6 years (range, 3 to 16 years). Among the adult participants, 61.6% self-identified as white. Age, race, and ethnicity information on pediatric participants is withheld, since it can be used to identify the participants. The timeline of stool collection is summarized in Fig. S1. Demographic information is summarized in Fig. S2 and Table S1.

### ToBRFV is more prevalent in human stool samples than PMMoV.

We tested whether RNA extracted from human stool samples was susceptible to RT-PCR inhibition. We assayed eight randomly selected RNA extracts for all three targets, the ToBRFV RdRP gene, the PMMoV CP gene, and the ToBRFV Mo gene, using both 1:10-diluted and undiluted templates. No RT-PCR inhibition was detected in the assay for the ToBRFV RdRP gene, since both diluted and undiluted templates provided the same results. However, in the assays for the PMMoV CP gene and the ToBRFV Mo gene, the diluted templates provided a higher concentration of the gene targets in one and two of the eight samples, respectively, indicating inhibition of the corresponding RT-PCRs. Since inhibition of RT-PCR was observed infrequently, in far less than 50% of the reactions, we assayed all of the samples in their undiluted format to retain higher sensitivity.

**FIG 4:**
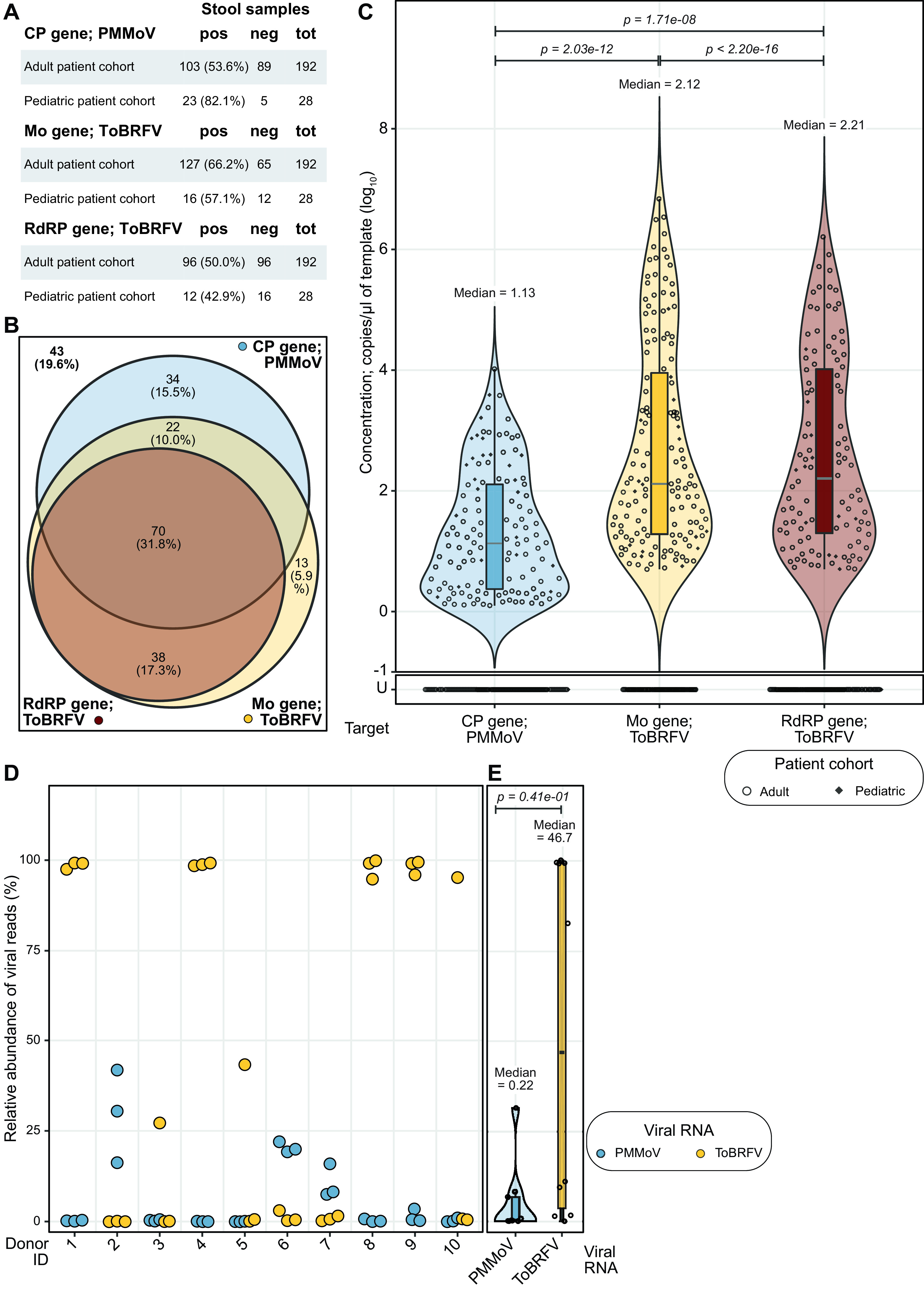
Prevalence of PMMoV and ToBRFV target genes in human stool samples. (A) Tabular summary of detection of the three gene targets in samples from adult and pediatric cohorts. The first column lists the name of the target gene and cohort, followed by the number and percentage of samples that were positive (pos) or number of samples that were negative (neg) for that target gene and the total number of samples tested (tot). (B) Venn diagram summarizing the detection of the PMMoV CP gene (blue) and the ToBRFV Mo (yellow) and RdRP (red) genes across 220 human stool samples. In 34 (15.5%), we detected only the PMMoV CP gene, while 13 (5.9%) had only the ToBRFV Mo gene. In 38 (17.3%) samples, we detected both ToBRFV target genes, while 22 (10.0%) had both the PMMoV CP gene and ToBRFV Mo gene. In 70 (31.8%) samples, we detected all three gene targets, while 43 (19.6%) had none of them. (C) Dot plot marking the concentrations of PMMoV CP (blue), ToBRFV Mo (red) and RdRP (yellow) genes, with violin and box plots summarizing their distributions, in RNA extracted from stool samples collected from humans. The *x* axis marks the target genes, and the *y* axis shows their concentrations. U, undetermined (samples with no detectable gene target above the LoB). The concentration of the PMMoV CP gene had a median of 1.13 with a standard deviation of 1.00 and IQR of 1.74 log_10_ copies/μL of template, the ToBRFV Mo gene had a median of 2.12 with a standard deviation of 1.69 and IQR of 2.67 log_10_ copies/μL of template, and the ToBRFV RdRP gene has a median of 2.20 with a standard deviation of 1.56 and IQR of 2.72 log_10_ copies/μL of template. *P* values derived from paired Wilcoxon signed-rank tests with continuity correction and excluding samples with undetermined concentration across all combinations of the three gene targets are listed at the top of the plot. (D) Dot plot marking the relative abundance of viral reads of PMMoV (blue) and ToBRFV (yellow) from previously published metatranscriptomics data derived from healthy stool samples. The *x* axis shows the 10 donors who provided samples, and each sample provided RNA sequences in biological triplicate; each dot denotes a single replicate. The *y* axis shows relative abundance. (E) Dot plot summarizing data from panel D, now including violin and box plots to highlight distribution of viral RNA concentrations and associated statistics. The *x* axis marks the target viral RNA, and the *y* axis shows their relative abundance in percent. Dots represent the averages of data from three biological replicates. PMMoV (blue) is present at a median relative abundance of 0.217% with a standard deviation of 9.83% and IQR of 5.19%, ToBRFV (yellow) is present at a median relative abundance of 46.7 with a standard deviation of 48.5% and IQR of 95.4%. The *P* value at the top was derived from a Wilcoxon signed-rank test of pairwise differences in relative abundance with continuity correction and excluding samples with undetermined concentration.

Of 222 RNA extracts derived from 129 participants, 220 had detectable BCoV RNA. This suggests that two of the RNA extractions failed; those samples were therefore excluded from further analysis, altering our study cohort size to 127 (123 adult; 4 pediatric). Among the remaining stool samples, 126/220 (57.3%) had detectable levels of the PMMoV CP gene, while 143/220 (65.0%) had the ToBRFV Mo gene and 108/220 (49.1%) had the ToBRFV RdRP gene; the ToBRFV Mo gene was the most prevalent target gene. This prevalence varied in the two patient cohorts (Fig. 4A); 127/192 (66.2%) stool samples from adult participants had detectable amounts of the ToBRFV Mo gene, more than in the case of PMMoV CP gene (103/192; 54.7%), but only 16/28 (57.1%) stool samples from pediatric patients had detectable amounts of the ToBRFV Mo gene, fewer than in the case of PMMoV CP gene (23/28; 82.1%).

In analyzing the prevalence of the three gene targets of interest in the stool samples, we detected all three gene targets in 70 (31.8%) of the samples, while we detected none of the three gene targets in 43 (19.6%) (Fig. 4B; Fig. S5). Notably, in 34 (15.5%) of the samples, we detected only the PMMoV CP gene, and in 13 (5.9%), we detected only the ToBRFV Mo gene. In all samples in which we detected the ToBRFV RdRP gene, we also detected the ToBRFV Mo gene. This analysis suggests that while the ToBRFV Mo gene is the most prevalent RNA-based marker of human stool, combining this with the detection of the PMMoV CP gene will provide the most coverage, more than 80.0% of stool samples.

Next, we analyzed the abundance of each of these gene targets in stool samples. The median detected concentration of the PMMoV CP gene is lower than that of the ToBRFV Mo gene (1.13 log_10_ copies/μL of template versus 2.12 log_10_ copies/μL of template; Wilcoxon signed rank test *P = *2.03e−12) and the ToBRFV RdRP gene (1.13 log_10_ copies/μL of template versus 2.21 log_10_ copies/μL of template; *P = *1.17e−8) (Fig. 4C). These stool samples were derived from participants undergoing different treatments for underlying hematologic disorders. Therefore, we investigated whether the nature of treatment was a confounding factor. Here, again, we found that the median abundances of both target genes from ToBRFV are higher than that of the PMMoV CP gene, even when the samples were separated by treatment cohort (Fig. S6A). Further, a paired comparison of target gene abundances validates the previous observation that all samples that tested positive for the ToBRFV RdRP gene also tested positive for the ToBRFV Mo gene (Fig. S6B).

While the concentration of the various gene targets has so far been reported in copies per microliter of template, we recognize that studies also measure molecular targets in units per gram (dry weight) of stool sample. Therefore, we chose five samples per cohort at random, dried two biopsy punches from each sample, and found that the mean percent (dry weight) in the samples from adults undergoing HCT treatment was 23.6% (range, 18.2 to 33.9%), that for adults undergoing CAR-T treatment was 27.5% (range, 22.2 to 31.6%), and that of pediatric patients undergoing induction chemotherapy was 32.4% (range, 23.8 to 40.3%). We used the average percent dry weight to convert gene target concentrations to copies per gram (dry weight) of stool samples in Fig. S6C. In brief, the median concentrations of ToBRFV RdRP and Mo genes were 6.45 and 6.32 log_10_ copies/g (dry weight) of stool, and that of the PMMoV CP gene was 5.36 log_10_ copies/g (Fig. S6C).

To determine if our findings are generalizable to applications beyond a cohort of patients, we looked at an alternate data set recently generated in our group that sequenced RNA from stool collected from 10 healthy individuals in triplicate and frozen (20). In this data set also, the relative abundance of ToBRFV was consistently greater than that of PMMoV, as reflected by their median relative abundances of 46.7 versus 0.22% viral RNA reads (Fig. 4D). Taken together, these observations indicate that the abundance of ToBRFV is greater than that of PMMoV in human stool samples, and the ToBRFV Mo gene may thus be preferable to the PMMoV CP gene as an MST marker.

### The ToBRFV Mo gene is prevalent and abundant in wastewater samples.

Wastewater is a complex matrix containing human stool and other biological excretions, in addition to food waste, industrial waste, and infiltrating stormwater in some cases. We next validated the molecular detection test developed here for testing this sample type. We acquired wastewater solid samples from 15 cities in the United States, extracted RNA, and assayed it for the presence and abundance of the gene targets of interest.

**FIG 5:**
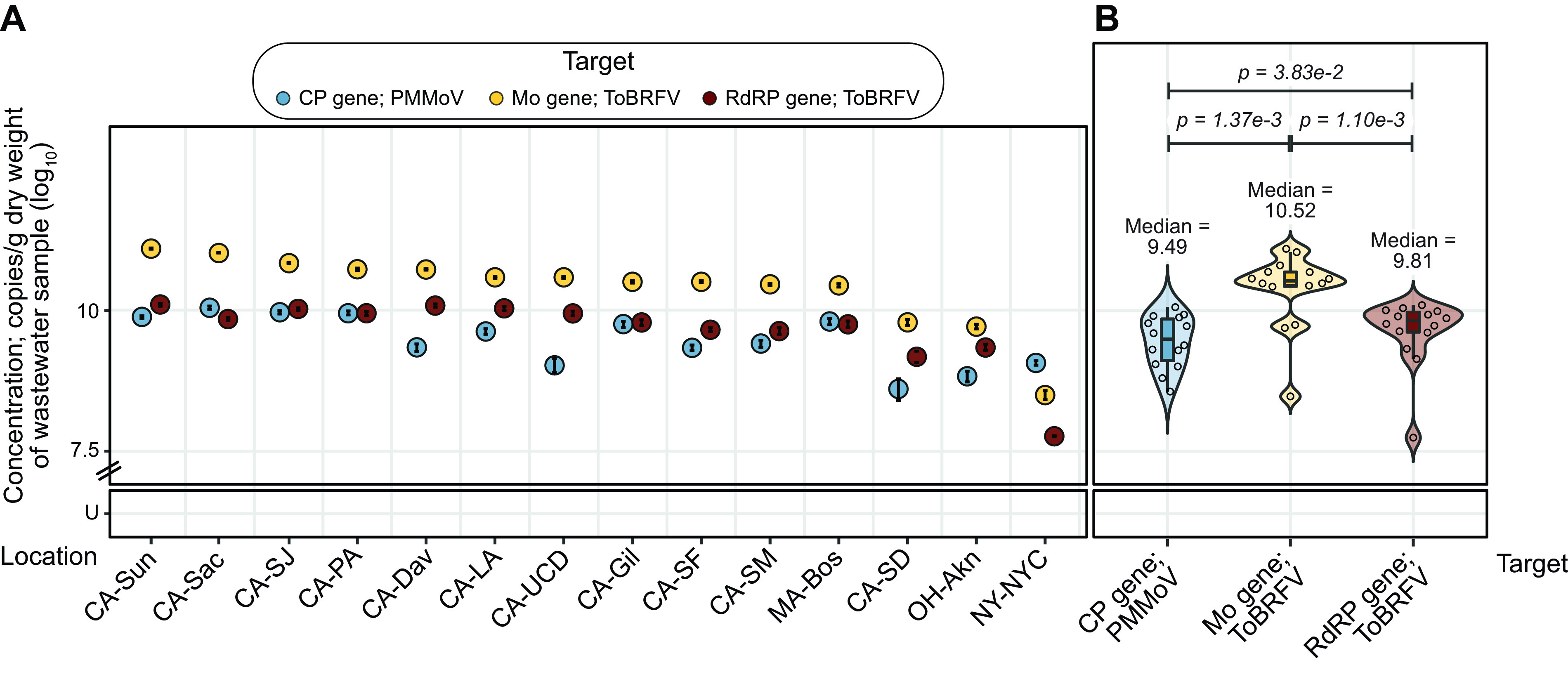
Concentrations of PMMoV and ToBRFV target genes in wastewater samples from across the USA (A) Dot plot marking the concentrations of PMMoV CP (blue), ToBRFV Mo (yellow), and ToBRFV RDRP (red) genes across samples. Error bars marking the standard deviation are plotted along with the dots and are mostly subsumed within the dots. The *x* axis shows the 15 cities from which samples were obtained, in decreasing concentration of the Mo gene; abbreviations for states and cities are expanded in Table S2. The *y* axis shows concentrations of the genes. (B) Dot plot marking the concentrations of PMMoV CP (blue), ToBRFV Mo (red), and RdRP (yellow) genes, with violin and box plots summarizing their distributions, in RNA extracted from wastewater samples collected from across the United States. The *x* axis marks the target genes, and the *y* axis shows their concentrations. The PMMoV CP gene has a median of 9.49 with a standard deviation of 0.46 and IQR of 0.74 log_10_ copies/g (dry weight) of wastewater sample, ToBRFV Mo gene has a median of 10.5 with a standard deviation of 0.67 and IQR of 0.26 log_10_ copies/g, and ToBRFV RdRP gene has a median of 9.81 with a standard deviation of 0.60 and IQR of 0.36 log_10_ copies/g. *P* values were derived from paired Wilcoxon signed-rank tests with continuity correction across all combinations of the three gene targets. U, undetermined (samples with no detectable gene target above the LoB).

The extracted RNA from Wisconsin did not have detectable amounts of any of the gene targets; this matches unpublished data generated using this sample by a different group, and this RNA was excluded from further analysis, reducing our sample size to 14. Thirteen of these samples had more ToBRFV Mo gene than the other two molecular markers, with the sample from New York being the exception, having the PMMoV CP gene in the highest concentration (Fig. 5A). Looking at the data in aggregate, the samples had a median concentration of 10.5 log_10_ copies/g (dry weight) of wastewater solids (standard deviation of 0.67 and interquartile range [IQR] of 0.26 log_10_ copies/g) of the ToBRFV Mo gene, followed by 9.81 log_10_ copies/g (standard deviation of 0.60 and IQR of 0.36 log_10_ copies/g) of the ToBRFV RdRP gene and 9.49 log_10_ copies/g (standard deviation of 0.46 and IQR of 0.74 log_10_ copies/g) of the PMMoV CP gene. Pairwise comparison of gene target concentrations across samples using the Wilcoxon signed-rank test revealed that the increased detection of the ToBRFV Mo gene is statistically significant in comparison to detection of the PMMoV CP gene (*P = *1.37e−3) and the ToBRFV RdRP gene (*P = *1.10e−3).

Our analytical workflow to purify RNA from wastewater samples has previously been shown to yield templates free of RT-PCR inhibitors (21). Additionally, we diluted RNA extracts from wastewater samples 1:10,000 prior to use as templates in ddPCR assays to detect the ToBRFV and PMMoV gene targets. This high dilution further mitigates the likelihood of RT-PCR inhibition.

### The ToBRFV Mo gene matches crAssphage ORF000024 as an indicator of fecal contamination of stormwater.

crAssphage ORF000024 is a well established human-associated microbial source tracking marker (12). We compared concentrations of PMMoV and ToBRFV RNA targets to those of this crAssphage DNA target in stormwater draining from urbanized watersheds in the Bay Area. crAssphage ORF000024 was previously quantified in these samples and was reported by Graham et al. (22).

**FIG 6:**
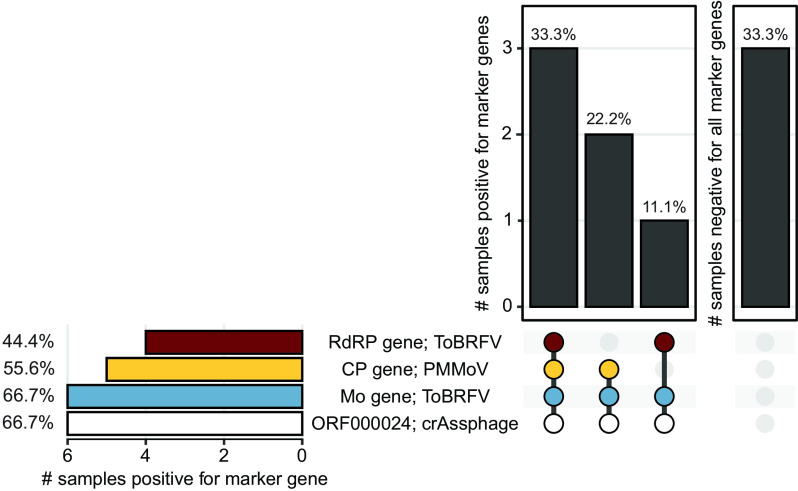
Prevalence of PMMoV, ToBRFV, and crAssphage target genes in stormwater samples from across California. UpSet plot summarizing the number of stormwater samples (total *n* = 9) that are either positive for multiple marker genes (left) or negative for all marker genes (right) in the vertical bar plots. Marker genes are listed under the plots, with colored dots representing presence and gray dots representing absence. Marker genes present in samples represented in the vertical bar are also connected by a thick line. The prevalence of independent marker genes is also summarized in the horizontal bar plot. All bars present data as percentages. Blue, PMMoV CP gene; yellow and red, ToBRFV Mo and RdRP genes, respectively; white, crAssphage ORF000024. Data for crAssphage were derived from a previous study (22).

We found that in the nine stormwater samples, crAssphage ORF000024 had the highest median concentration of 4.65, with a standard deviation of 0.56 and IQR of 0.66 log_10_ copies/L of stormwater, followed by the ToBRFV RdRP gene, with a median of 3.48, standard deviation of 0.97, and IQR of 1.24 log_10_ copies/L of stormwater, the ToBRFV Mo gene, with a median of 3.34, standard deviation of 0.99, and IQR of 1.36 log_10_ copies/L of stormwater, and finally the PMMoV CP gene, with a median of 3.02, standard deviation of 0.54, and IQR of 0.44 log_10_ copies/L of stormwater (Fig. S7). Pairwise comparison of gene target concentrations across samples using the Wilcoxon signed-rank test revealed that differences in concentrations are not statistically significant and gene targets are similarly abundant. The concentration of gene targets in each of the samples is presented in Fig. S7. Notably, the ToBRFV Mo gene was detected in as many samples (6/9) as crAssphage ORF000024 (Fig. 6). This result suggests that using an RNA-based marker from ToBRFV to detect human stool contamination of stormwater may be as useful as using the DNA marker from crAssphage ORF000024.

We obtained concentrations of PMMoV and ToBRFV gene targets using templates that were diluted 1:10. Higher dilutions led to lower detection of these gene targets. Therefore, we believe that the results reported here are free of influence from RT-PCR inhibition.

### Conclusions and limitations.

In this study, we generated eight nearly complete genomes of ToBRFV from wastewater and stool from the Bay Area. We catalogued SNPs in all existing genomes, including in those that we assembled here, and noted variations in viral genomes isolated from the same individual over ~100 days. We then went on to identify two sets of primers and probes that can universally detect ToBRFV across the world.

Assays developed using these primer and probe sequences are sensitive and specific for human stool and wastewater, as they were present in a wide range of wastewaters and stool samples and not present in any tested animal stool aside from one sample from a cat and another from a pig. Like the established viral MST target PMMoV (8), the ToBRFV target is derived from the genome of a plant virus likely present in the human stool owing to dietary intake of diseased plants. Concentrations of ToBRFV Mo and RdRP gene targets were as high as or higher than those of the PMMoV CP gene in wastewater and stormwater known to contain sewage. The high concentrations of ToBRFV targets in wastewater, as well as in human stool samples, suggest that they may be useful as endogenous fecal-strength controls for wastewater-based epidemiology applications (23), as well as an endogenous positive extraction control during nucleic acid extractions in studies seeking to quantify rare infectious-disease targets (8) in human stool.

Notably, we took a number of actions in our analytical workflow to guard against the inhibition of RT-PCRs by substances that can coelute with the nucleic acid templates. First, we purified all nucleic acids using commercial kits that are known to remove such inhibitors. In the case of wastewater samples, we acquired templates from a previous study that additionally employed an inhibitor removal kit (21). Second, we used ddRT-PCR, which is less sensitive to inhibition than RT-qPCR (24, 25). Finally, we diluted the nucleic acid templates used in the ddRT-PCRs to mitigate the effect of inhibitors. In instances where the nucleic acid concentration was low, we report data from undiluted templates and used the diluted templates to assess the presence of inhibitors. Overall, we identified little evidence of inhibition.

There are several limitations to this work. First, the specificity of the ToBRFV Mo and RdRP gene targets was tested using just one representative sample each of various non-human animal stools. Additional work to test more animal stool samples would be helpful to further characterize the assays’ specificity for human stool. Second, the sensitivities of the various assays were tested using human stool samples only from individuals residing in the Bay Area. It is possible that the distribution of the targets in individuals from other locations may differ from those studied here, and more work to document the ToBRFV prevalence and abundance in samples globally is encouraged. Third, it is possible that the extraction methods used to acquire nucleic acids from the various samples may have biases for the gene targets assayed in this study. Since ToBRFV and PMMoV both belong to the genus *Tobamovirus*, we believe that they are likely treated similarly by the extraction methods. However, in the stormwater samples, we compared gene targets from these viruses with those from crAssphage. crAssphage may react differently in the extraction process, and such variations are yet to be studied. Fourth, differences in the storage conditions of samples used in this study may have influenced our results (26). For instance, we have found that freezing and thawing samples can influence viral quantification, while differences in the duration of sample storage have a negligible effect (27). Notably, all frozen samples used in the current study underwent only one freeze-thaw cycle for this project. However, samples were stored for different durations, and this may have impacted our results in ways we cannot quantify. Biobanking of samples is a vital step in this research, and inherent variations in duration of storage are unavoidable. Fifth, we assayed wastewater solids sampled from around the United States, from New York to California, and they contained high concentrations of the ToBRFV targets. Further work using samples from around the world will be valuable to testing the generalizability of the assays. Notably, the presence of ToBRFV genomes from this study and others collected from many countries reassures us that ToBRFV is likely to be a universal global MST marker. Finally, as more ToBRFV genomes become available, it will be important to test whether the primers and probes developed herein continue to overlap conserved regions of the genomes.

## MATERIALS AND METHODS

### Assembly and analysis of ToBRFV genomes and design of hydrolysis probe RT-PCR assays.

In order to design ToBRFV-specific primers and probes for hydrolysis-probe RT-PCR assays, all ToBRFV genomes available in February 2021 were obtained. These were supplemented with new genomes assembled from stool samples processed and sequenced in this study (Table 2).

In February 2021, all nearly complete genomes (*n* = 70) of ToBRFV were downloaded from NCBI GenBank. In the same month, raw reads from the only publicly available wastewater metatranscriptomics data set (obtained from wastewater in the Bay Area, collected between May and July 2020; BioProject accession no. PRJNA661613) were also downloaded. Using these reads, five ToBRFV genomes were assembled as outlined below.

In addition to using existing sequencing data and genomes, RNA from three human stool samples obtained longitudinally from one individual were also sequenced; the first two samples were collected 10 days apart, and the third was collected 93 days after the second sample. The samples were obtained from an individual with laboratory-confirmed COVID-19 and were collected under an Institutional Review Board (IRB)-approved protocol (Stanford IRB protocol 55619). Total RNA was extracted from these samples, rRNA was depleted, and libraries were prepared and sequenced using NextSeq 550 as outlined in the supplemental material.

The following bioinformatic methods were used to assemble genomes from both the existing (from wastewater) and newly obtained (from stool) metatranscriptomics reads. Reads were trimmed with Trim Galore (version 0.4.0) using Cutadapt (version 1.8.1) (28) set to flags –q 30 and –illumina. SPAdes (version 3.14.1) set to -meta was used to assemble genomes *de novo* (28, 29). Contigs belonging to ToBRFV were classified using One Codex (30). Genes were annotated using Prodigal (version 2.6.3) set to -meta (31). If all genes were predicted on the negative strand of the contig, the entire contig was reverse complemented. The completeness of potential ToBRFV genomes was assessed using CheckV (version 1.0.1) (32), and genomes that were >90.0% complete were selected for subsequent analyses.

To assess strain diversity of ToBRFV in the longitudinal stool samples, RNA sequencing reads from stool samples were aligned to the ToBRFV reference genome (NCBI accession no. NC_028478) using Bowtie (version 2.4.2) (33). The resulting bam files were used as input into inStrain (version 1.0.0) (34) to calculate population-level average nucleotide identity (popANI) between genomes.

To assess abundance of ToBRFV relative to other viruses in the RNA sequencing (RNA-Seq) data, reads were classified against the Viral Kraken2 database (https://benlangmead.github.io/aws-indexes/k2) (35) using default parameters. Counts from the classification were used to calculate relative abundance of viral reads.

A multiple-sequence alignment of all nearly complete genomes of ToBRFV, including genomes downloaded from NCBI GenBank in February 2021 (70 genomes) and those we assembled from wastewater and stool (8 genomes), was performed using Geneious Alignment (Geneious Prime version 2021.0.3) (36) with default settings, global alignment with free end gaps, and cost similarity matrix set to 65.0%. SNPs were called from the multiple sequence alignment using SNP-Sites (version 2.5.1) (37). A phylogenetic tree was built using Geneious Tree Builder (version 2021.0.3) with default settings and a Tamura-Nei genetic distance model with the neighbor-joining method. Primers and probes were designed to be specific for ToBRFV using Geneious Primer (version 3 2.3.7) (38) based on the 78 genomes we had access to in February 2021 with near-default settings, requiring product size to be between 95 and 125 bp and primers to be based on the consensus with 100.0% identity across all ToBRFV genomes. Primers and probe sequences were screened for specificity, *in silico*, using NCBI BLAST.

### New genomes available in November 2022.

New ToBRFV genomes became available on public databases between the first phase of this study in February 2021 and the completion of this work in November 2022. Specifically, an additional 113 genomes were downloaded from NCBI, bringing the total to 183 (39) and 250 assembled ToBRFV genomes from a study of wastewater from Southern California (18) were downloaded (Table 2).

As Geneious alignment and tree building are computationally intensive, a phylogenetic tree of all 441 nearly complete genomes of ToBRFV was built using ViPTree (40) and visualized and color coded by region using Iroki (41). In addition, the applicability of the primers and probes designed in this study was tested *in silico* using NCBI BLAST.

### Processing of animal stool samples for RNA quantification.

One stool sample each was collected from (i) a single animal (cat, dog, horse, pig, and rabbit) raised as a pet, (ii) a group of cohabiting animals of a single kind (chicken, cow, goat, mouse, and sheep) from Deer Hollow Farms (California, USA), (iii) a group of cohoused ducks and geese at Deer Hollow Farms, and (iv) wild animals (bear and deer). Samples were collected in a sterile clinical stool collection container by individuals wearing gloves and using a spatula. Samples were transported at room temperature, aliquoted into cryovials, and stored at −80°C within 12 h from collection. Samples were further processed within a month of storage and did not go through any freeze-thaw cycles prior to the current work.

A single, defined solid volume of sample of each animal stool was acquired using Integra Miltex biopsy punches with a plunger system (Thermo Fisher Scientific; catalog no. 12-460-410) and placed in independent microcentrifuge tubes. Five hundred microliters of RNAlater (Ambion; catalog no. AM7023M) was added, and samples were processed using a previously validated methodology (24) as follows. A stock BCoV vaccine was prepared by adding 3 mL of 1× phosphate-buffered saline (PBS; Fisher Scientific; catalog no. BP399-500) to one vial of lyophilized Zoetis Calf-Guard bovine rotavirus-coronavirus vaccine (catalog no. VLN 190/PCN 1931.20) to create an undiluted reagent as per the manufacturer’s instructions. Ten microliters of this attenuated BCoV vaccine was added to every sample as an external control and vortexed for 15 min. BCoV is an RNA virus that was previously found to be a reliable positive control for RNA extraction from stool (24). Samples were processed immediately after addition of the BCoV control.

### Collection and processing of human stool samples used for RNA quantification.

Human stool samples were previously collected and biobanked in RNAlater solution as part of Stanford Institutional Review Board-approved protocols 8903 (Blood and Bone Marrow Grafting for Leukemia and Lymphoma), 11062 (Genome, Proteome and Tissue Microarray Studies in Childhood malignant and Non-Malignant Hematologic Disorders), and 48548 (Hematopoietic Recovery During Induction Chemotherapy in Pediatric Leukemia). From these biobanks, 194 and 28 samples collected from 125 adult and 4 pediatric participants, respectively, from November 2019 to October 2020 were used in this study. These samples had been stored for between 1 and 12 months depending on the date of collection and did not go through any freeze-thaw cycles prior to the current work. All samples were spiked with 10 μL of attenuated BCoV vaccine as a control and processed similarly to the animal stool samples.

### RNA extraction from all stool samples used for RNA quantification.

RNA was extracted from stool samples using the QIAamp viral RNA minikit (Qiagen; catalog no. 52906) as previously optimized (24). Briefly, the prepared stool samples were spun down at 10,000 × *g* for 2 min to acquire 140 μL of clarified supernatant. RNA was extracted from this supernatant using the QIAamp viral RNA minikit (Qiagen; catalog no. 52906) as per the manufacturer’s instructions. Finally, RNA was eluted in 100 μL of the elution buffer and stored in a 96-well plate at −80°C for up to 12 months. Notably, in previous work on BCoV and SARS-CoV-2 RNA (24), we found that RNA extracted using this methodology was free of RT-PCR inhibitors.

### Augmenting analysis of stool with metatranscriptomic data from healthy individuals.

As described below, we assessed the prevalence and abundance of MST markers in stool acquired from participants with hematologic disorders. This presented an obstacle to the generalizability of our work. Therefore, we acquired metatranscriptomics data from stool samples from 10 healthy participants presented in a previous study (20). Though many human stool metatranscriptomic data sets exist, this was the most recent data set we had access to.

### Collection and processing of wastewater samples used for RNA quantification.

Settled solids were obtained from 15 wastewater treatment plants across the United States (Table S2). Solids were collected from the primary clarifier or settled from a 24-h composited influent sample using Imhof cones. Samples were collected in sterile containers and transported to the lab. Samples from the Bay Area were processed immediately, while other samples were stored at −80°C until analysis (between 5 and 20 months). None of the samples stored at −80°C underwent a freeze-thaw cycle prior to the current work.

Solids were dewatered using centrifugation, and then an aliquot of the dewatered solids was set aside for dry-weight analysis. Solids were then suspended in a buffer (approximately 75 mg/mL), homogenized, and centrifuged. This suspension of solids in buffer was found to alleviate inhibition of RT-PCR (21). An aliquot of the supernatant was processed for total nucleic acid extraction using Chemagic 360 (Perkin Elmer). Nucleic acid preparations from wastewater samples are known to contain PCR inhibitors that interfere with their accurate quantification using PCR-based methods. Therefore, inhibitors were removed using the OneStep PCR inhibitor removal kit (Zymo Research; catalog no. D6035), yielding nucleic acids in 50 μL of eluant. These methods have been published in detail (42), and step-by-step protocols are available on protocols.io (43, 44).

### Source of RNA extracted from stormwater samples used for RNA quantification.

RNA extracted from stormwater samples was derived from a previous study from our group (22). Briefly, nine stormwater samples from the Bay Area—one each from the Guadalupe River, Pilarcitos Creek, San Francisquito Creek, and San Pedro Creek, two from Stevens Creek, and three from Lobos Creek—collected between October 2018 and March 2019 were used to extract RNA (Table S3). Specifically, stormwater samples were collected in the winter of 2018-2019, and immediately upon collection, viruses were concentrated from 1 to 5.5 L of stormwater using electronegative filtration using 0.05 M MgCl_2_. The filtration membranes were preserved in 250 μL of RNAlater (Qiagen; catalog no. 76104) for 5 min prior to storage at −80°C.

Nucleic acids were extracted into 100 μL of RNase-free water from the stored filtration membrane with a Qiagen DNA/RNA AllPrep PowerViral kit using a protocol including β-mercaptoethanol and bead beating and stored as aliquots in microcentrifuge tubes at −80°C. Extraction of nucleic acids was completed within 6 months of sample collection. Samples were thawed on ice prior to use in crAssphage assays. Separate additional frozen aliquots of extracted nucleic acids that had not undergone any freeze-thaw cycles were stored at −80°C and used in the current work. Previous work suggested that RT-PCR inhibitors from the samples were not coextracted in this RNA extraction process (22). These extracts were used in the current study after 30 months of storage.

### Quantification of viral RNA sequences by ddRT-PCR.

The CP gene encoding the coat protein from PMMoV, the genes encoding the movement protein (Mo) and RNA-dependent RNA polymerase (RdRP) from ToBRFV, and the gene encoding the membrane (M) protein from BCoV were quantified using ddRT-PCR. We chose ddRT-PCR instead of RT-qPCR for nucleic acid detection and quantification because of its superior sensitivity and resistance to PCR inhibitors (24, 25).

Templates derived from non-human animal stool were assayed for the PMMoV and ToBRFV gene targets in their undiluted and 1:10 diluted formats. Templates derived from human stool were assayed for the PMMoV and ToBRFV gene targets in their undiluted format. However, 30 of these templates yielded ddRT-PCRs where all the droplets were positive. This is not ideal, because ddRT-PCRs rely on a Poisson distribution of the template across droplets to accurately quantify gene targets. Therefore, these templates were diluted 1:10,000 and reassayed for the relevant gene targets. Additionally, templates from eight samples were randomly chosen, diluted 1:10, and assayed for the PMMoV and ToBRFV gene targets to detect any inhibitors. Templates derived from wastewater samples were diluted 1:10,000 before assaying for PMMoV and ToBRFV gene targets. In cases where these gene targets were undetectable at this high dilution, we assayed templates at 1:100 and 1:10 dilutions and in an undiluted format. Templates derived from stormwater samples were assayed for the PMMoV and ToBRFV gene targets at three dilutions: 1:10,000, 1:1,000, and 1:10. Results reported are from the 1:10 dilution, since the gene targets were undetectable at higher dilutions.

Human participants in this study were enrolled and hospitalized during the first year of the COVID-19 pandemic. We tested their stools for genes encoding the envelope (E) and a nucleocapsid (N2) protein from the SARS-CoV-2 genome as previously described (24), in order to assess occurrence of COVID-19 during hospitalization at Stanford Hospital. However, we did not find any presence of COVID-19 RNA in these samples. Sequences of the newly designed primers and probes targeting ToBRFV Mo and RdRP genes are listed in Table 1. Previously published primers and probes targeting BCoV, PMMoV, and SARS-CoV-2 RNAs are listed in Table S4.

The droplet digital PCR application guide for QX200 machines (Bio-Rad) (45) and digital minimum information for publication of quantitative real-time PCR experiments (dMIQE) guidelines (46) inform this methodology. The experimental checklist recommended by dMIQE is available at the Stanford Digital Repository (https://purl.stanford.edu/nf771cs9443). A Biomek FX liquid handler (Beckman Coulter) was used to prepare the ddRT-PCR by adding 5.5 μL of eluted RNA to 5.5 μL supermix, 2.2 μL reverse transcriptase, 1.1 μL of 300 nM dithiothreitol (DTT), 1.1 μL of each of the 20× custom ddPCR assay primer-probe mixes (Bio-Rad; catalog no. 10031277), and 5.5 μL of nuclease-free water (Ambion; catalog no. AM9937, lot 2009117). The supermix, reverse transcriptase, and DTT were from the one-step ddRT-PCR Advanced kit for probes (Bio-Rad; catalog no. 1864021). A QX200 AutoDG droplet digital PCR system (Bio-Rad) was used to partition the samples into droplets of roughly 1 nL using the default settings, and the template was amplified using a Bio-Rad T100 thermocycler with the following thermocycling program: 50°C for 60 min, 95°C for 10 min, 40 cycles of 94°C for 30 s and 55°C for 1 min, followed by 1 cycle of 98°C for 10 min and 4°C for 30 min with a ramp speed of 1.6°C per s at each step (47).

A multistep approach was adopted to calculate the raw RNA concentrations, as previously described (24). Every plate in the ddRT-PCR assays included appropriate positive and negative controls, including synthetic target genes (PMMoV CP gene, and ToBRFV Mo and RdRP genes) cloned in the pIDT vector, RNA extracted from reconstituted attenuated BCoV vaccine, water, and RNAlater. The signal threshold corresponding to every plate was manually set between the mean positive and negative amplitudes of these controls such that the number of detected copies in the negative controls was minimal and those from the relevant positive controls most closely matched the expected RNA concentration. Next, the difference between the mean negative amplitude and the threshold amplitude in the negative-control reactions was calculated and added to the mean negative amplitude for every sample on that plate. Applying this threshold yielded the raw RNA concentrations.

In order to derive the limit of blank (LoB) and limit of detection (LoD) of our assays to further process the raw RNA concentrations we adopted the following steps. (i) The LoB indicates the highest background RNA concentration registered from control samples that are confidently negative for the relevant gene targets. In order to determine the LoB, water, RNAlater, and synthetic genes discordant with the target gene (e.g., the ToBRFV Mo gene is a negative control in an assay of the ToBRFV RdRP gene) were assayed in duplicate. The highest RNA concentration measured in these LoB samples for each of the primer/probe sets was set as the relevant LoB. All samples in which we detected an RNA concentration equal to or less than the LoB were set to zero. (ii) The LoD is defined as the lowest concentration of RNA that can be reliably detected. To determine the LoD, duplicate serial dilution series of the synthetic target genes at 1, 2, 5, 10, 100, and 1,000 copies/μL of template were assayed for the corresponding target gene (Fig. S1). The synthetic target genes were acquired from Integrated DNA Technologies and cloned in their standard backbone, pIDTSmart. These plasmids were transformed into E. coli, isolated using the QIAprep Spin miniprep kit (Qiagen; catalog no. 27104) and quantified using Qubit. The LoD for a primer/probe set was defined as the lowest concentration of the standard at which both replicates had a detectable RNA concentration. All viral RNA concentrations below the LoD were set to zero.

Finally, after these data processing and analysis steps, the samples were assigned a final viral RNA concentration in copies per microliter of template. “Eluate” refers to the 100 μL of sample acquired from the RNA extraction. Viral RNA concentrations from animal and human stool samples are expressed in copies per microliter of template, those from wastewater samples are in copies per gram of wastewater, and those from stormwater samples are in copies per liter of stormwater.

In the case of all non-human animal stool, wastewater and stormwater samples, RNA was quantified using singleplex reactions. For the human stool samples, which were limited in quantity, the detection of the BCoV M gene and PMMoV CP gene were multiplexed with the detection of the SARS-CoV-2 E and N2 genes using orthogonal fluorescent probes. After extensive optimization (outlined in the supplemental material), we paired the detection of the E gene (SARS-CoV-2) with that of the CP gene (PMMoV) and detection of the N2 gene (SARS-CoV-2) with that of the M gene (BCoV) in two independent reactions using the carboxyfluorescein (FAM) and hexachlorofluorescein (HEX) fluors, respectively.

### Data analysis and generation of plots.

Data were analyzed using RStudio (v 1.2.5042), using the packages cowplot (v 1.1.1), dplyr (v 1.0.8), eulerr (v 6.1.1), ggplot2 (v 3.3.6), and UpSetR (v 1.4.0).

### Data availability.

Newly generated genomes and raw sequencing reads from stool samples are available on NCBI’s Sequence Read Archive (SRA) database under accession no. PRJNA917455. All other relevant data are included in the article and available in the Stanford Digital Repository (https://purl.stanford.edu/nf771cs9443).
