# Supplementary material for: The Tomato Brown Rugose Fruit Virus Movement Protein Gene Is a Novel Microbial Source Tracking Marker
Source: Appl Environ Microbiol. 2023 Jul 5;89(7):e00583-23. doi: 10.1128/aem.00583-23 (PMC10370318; doi:10.1128/aem.00583-23)
Supplement: Supplemental file 1 — Supplemental material. Download aem.00583-23-s0001.docx, DOCX file, 5.2 MB [file aem.00583-23-s0001.docx]

**Supplemental Materials**

Tomato brown rugose fruit virus Mo gene is a novel microbial source tracking marker

Aravind Natarajan*^1,2^, Brayon J. Fremin*^1^, Danica T. Schmidtke^3^, Marlene K. Wolfe^4^, Soumaya Zlitni^1,2^, Katherine E. Graham^4#^, Erin F. Brooks^2^, Christopher J. Severyn^5^, Kathleen M. Sakamoto^5^, Norman J. Lacayo^5^, Scott Kuersten^6^, Jeff Koble^6^, Glorianna Caves^6^, Inna Kaplan^7^, Upinder Singh^8^, Prasanna Jagannathan^8,9^, Andrew R. Rezvani^7^, Ami S. Bhatt^1,2,**^, Alexandria B. Boehm^4,^**

**Aravind Natarajan and Brayon J Fremin contributed equally to this work. Order of co-first authors was determined by contribution to writing the manuscript.*

# Affiliations

^1^ Department of Genetics, Stanford University, Stanford, CA, U.S.A.

^2^ Department of Medicine (Hematology, Blood and Marrow Transplantation), Stanford University, Stanford, CA, U.S.A.

^3^ Department of Microbiology and Immunology, Stanford University, Stanford, CA, U.S.A.

^4^ Department of Civil and Environmental Engineering, Stanford University, Stanford, CA, U.S.A.

^5^ Department of Pediatrics, (Hematology/Oncology/Stem Cell Transplant & Regenerative Medicine), Stanford University, Stanford, CA, U.S.A.

^6^ Illumina

^7^ Department of Medicine (Blood and Marrow Transplantation and Cellular Therapy), Stanford University, Stanford, CA, U.S.A.

^8^ Department of Medicine (Infectious Diseases and Geographic Medicine), Stanford University, Stanford, CA, U.S.A.

^9^ Department of Microbiology and Immunology, Stanford University, Stanford, CA, U.S.A.

# Present address

^#^ Katherine E. Graham: School of Civil and Environmental Engineering, Georgia Institute of Technology, Atlanta , GA 30332, U.S.A.

# Corresponding authors

**

Ami S. Bhatt, Center for Clinical Sciences Research RM. 1155b, Stanford University, Stanford, CA, 94305. Tel: (650) 498-4438; Email: [asbhatt@stanford.edu](mailto:asbhatt@stanford.edu).

Alexandria B. Boehm, Jerry Yang & Akiko Yamazaki Environment & Energy Building RM. 189, Stanford University, Stanford, CA 94305. Tel: (650) 724-9128; Email: [aboehm@stanford.edu](mailto:aboehm@stanford.edu).

**Contents**

[**Affiliations**](#_innb2zy09fph) 1

[**Present address**](#_s1dt4fjq43xs) 1

[**Corresponding authors**](#_98fnabgev4yk) 1

[**Supplemental figures**](#_wh1vrmphhgmi) 3

[Figure S1. Timing of stool collection from human participants undergoing treatment for hematological disorders.](#_9cmnqwoncnpb) 4

[Figure S2. Summary demographics of participants undergoing treatment for hematological disorders who provided stool samples for this study.](#_kv34qdzdugro) 5

[Figure S3. Phylogenetic tree of 441 near complete genomes of ToBRFV, including eight genomes generated in the current study from wastewater and stool](#_fwxtsj7ym23o) 6

[Figure S4. Analysis of specificity and sensitivity of primer/probes targeting genes in PMMoV and ToBRFV.](#_6x1r0oz5f40f) 8

[Figure S5. Prevalence PMMoV and ToBRFV target genes in human stool samples detected by ddRT-PCR.](#_1htv48tupur1) 9

[Figure S6. Concentrations of PMMoV and ToBRFV target genes in human stool samples detected by ddRT-PCR.](#_6y682yh4tubr) 11

[Figure S7. Concentrations of PMMoV, ToBRFV and crAssphage target genes in stormwater samples from across California.](#_2ri519vs3xgq) 12

[Figure S8. 1D amplitude of ddRT-PCR assays testing compatibility of primer/probes for multiplexed assays.](#_84vlls3s7yhr) 14

[**Supplemental table**](#_2j9jlzxejvke) 15

[Table S1. Demographic distribution of participants who provided stool for ddRT-PCR](#_y469ed5hmbgi) 15

[Table S2. Information on wastewater samples.](#_91yvyo9wrn7w) 17

[Table S3. Information on stormwater samples from California](#_ai1xwt77icsf) 18

[Table S4. Sequences of oligonucleotides used as primers and probes](#_ierhi21grrs4) 19

[Table S5. Assessment of newly assembled ToBRFV genomes](#_gs3rnqme89qj) 20

[Table S6. Summary of template concentrations from all tested multiplexed reactions](#_rqd4fndyeief) 21

[**Supplemental notes**](#_3h0y0vgtlnv8) 24

[Note S1. Sequencing of total RNA from three stool samples.](#_r3593shgtnqa) 24

[Note S2. Identifying primers/probes that are compatible in multiplexed ddRT-PCR assays](#_e0fug86bbiqx) 26

[**References**](#_j3ijx8sybzlu) 29

####

#

#
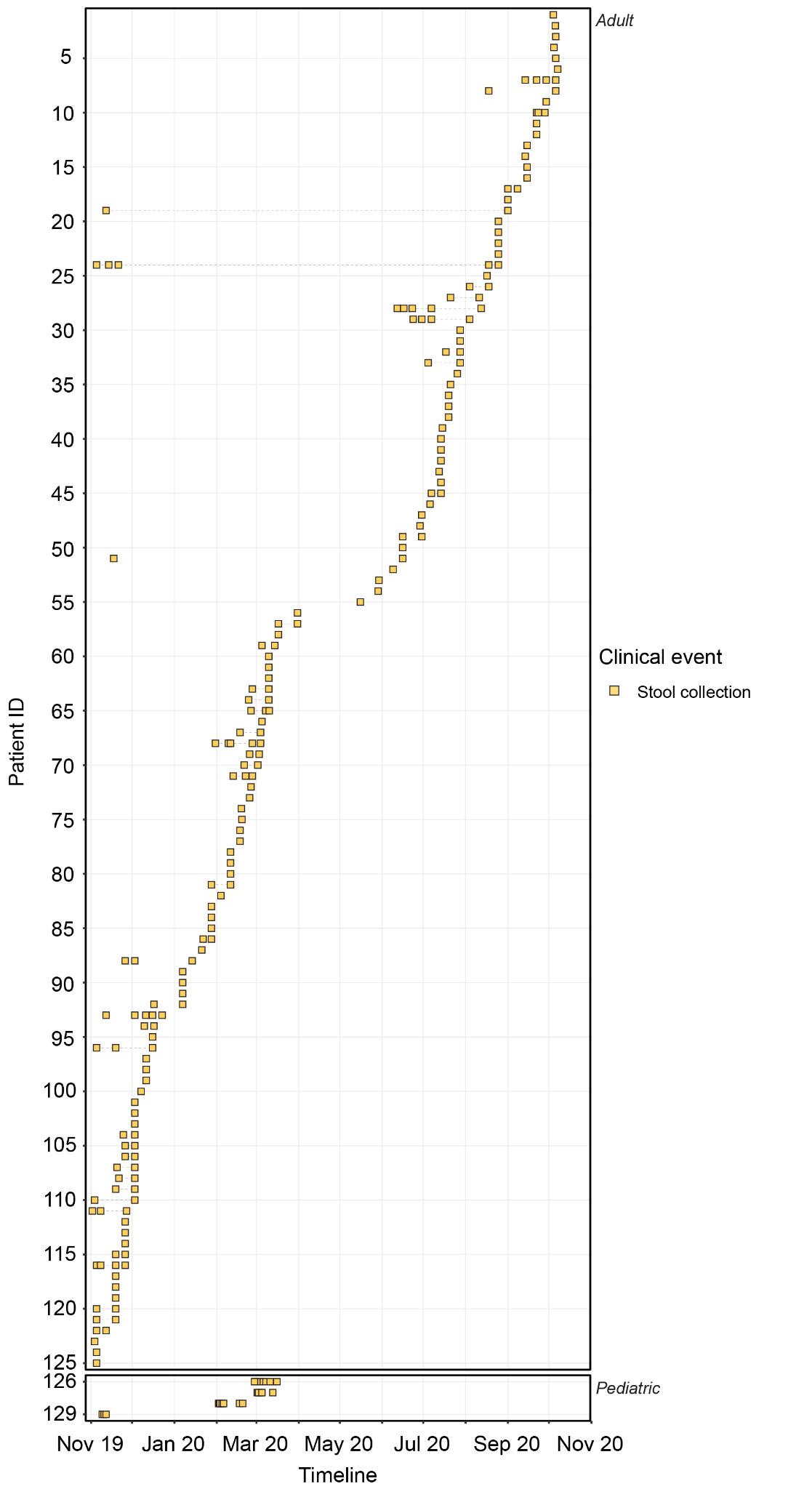
Supplemental figures

## Figure S1. Timing of stool collection from human participants undergoing treatment for hematological disorders.

The timeline from the start of the study in November 2019 to November 2020 is plotted on the x-axis. Every participant in the cohort is represented by an anonymized study ID on the y-axis and a corresponding horizontal line across the plot. 125 adult participants are represented in the upper panel and 4 pediatric participants are represented in the lower panel. Dates of stool collected are marked by a yellow square.

#

##
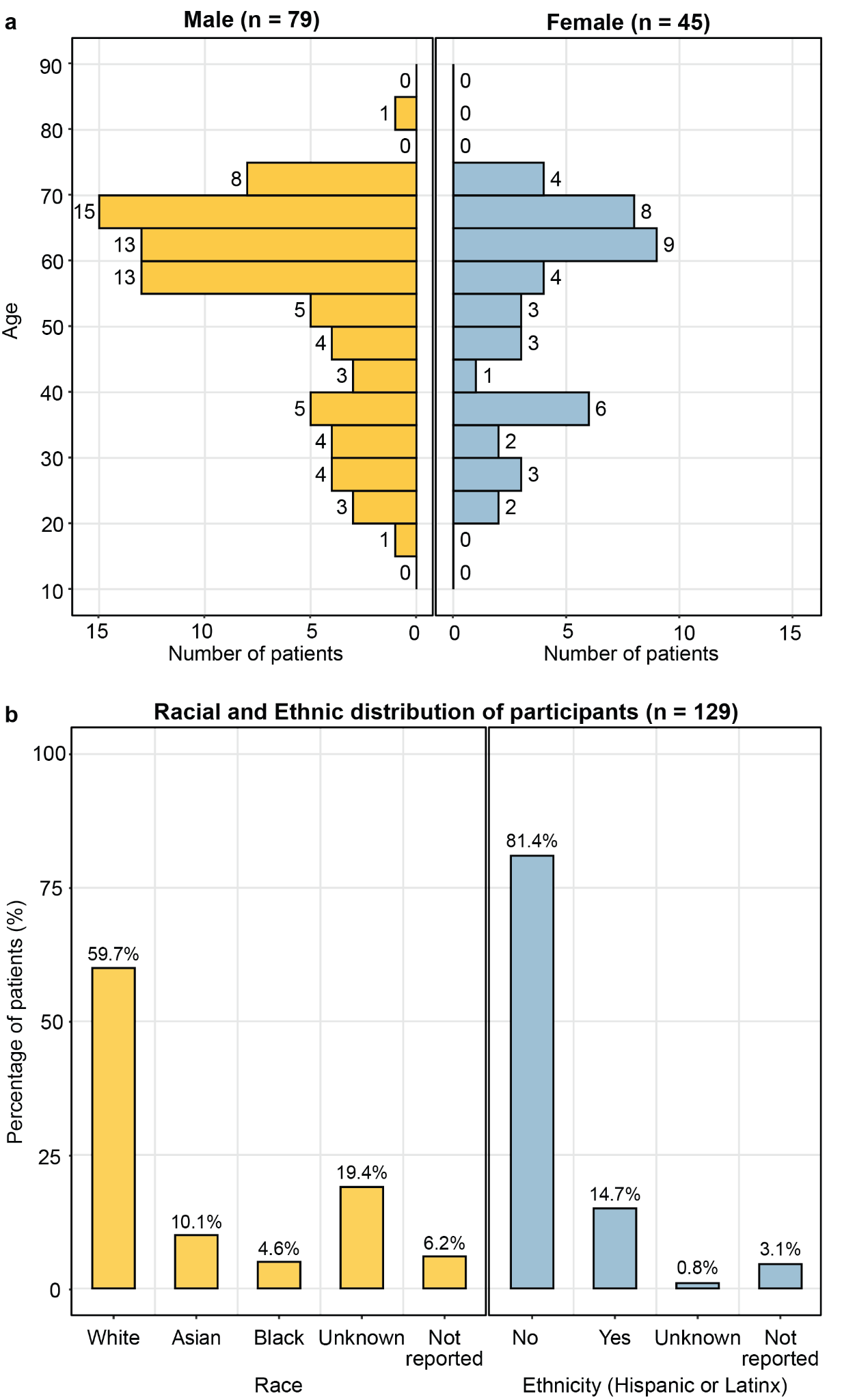
Figure S2. Summary demographics of participants undergoing treatment for hematological disorders who provided stool samples for this study.

a. Age distribution of 124 adult participants, 79 male and 45 female. The x-axis lists the number of participants and the y-axis lists age ranges at 10-year intervals. The number of participants in each category (range = 5 years) is listed at the head of the relevant bars. This data does not include 1 adult participant who did not provide their age and 4 pediatric participants whose ages are not listed to preserve anonymity. b. Racial and ethnic distribution of 129 adult and pediatric participants. The x-axis lists race or ethnicity categories and the y-axis lists the percentage of participants. The percentage of participants in each category is listed at the top of the relevant bars. Not reported refers to data that is aggregated in order to avoid information that can be used to identify participants. Associated cumulative data are summarized in Table S1.

##


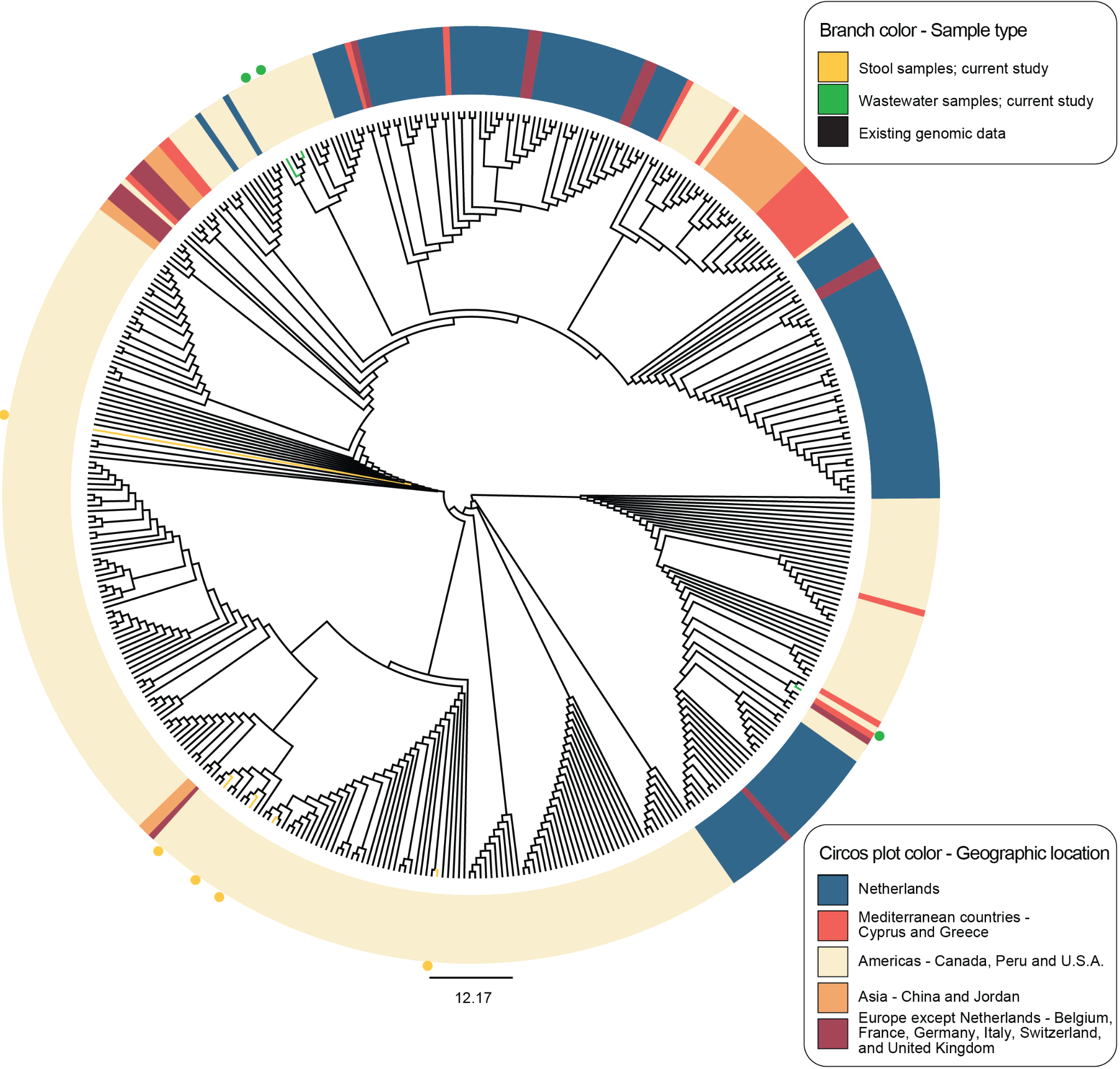


## Figure S3. Phylogenetic tree of 441 near complete genomes of ToBRFV, including eight genomes generated in the current study from wastewater and stool

433 preexisting genomes are represented by black, five genomes derived from wastewater samples by yellow, and three by green branches and corresponding dots in the outermost ring. The outer circle highlights the geographic location of the source of the genomes, where blue marks samples from Netherlands, salmon from Mediterranean countries (Cyprus and Greece), cream from Americas (Canada, Peru and U.S.A.), orange from Asia (China and Jordan), and brown from Europe except Netherlands (Belgium, France, Germany, Italy, Switzerland, and United Kingdom).

##
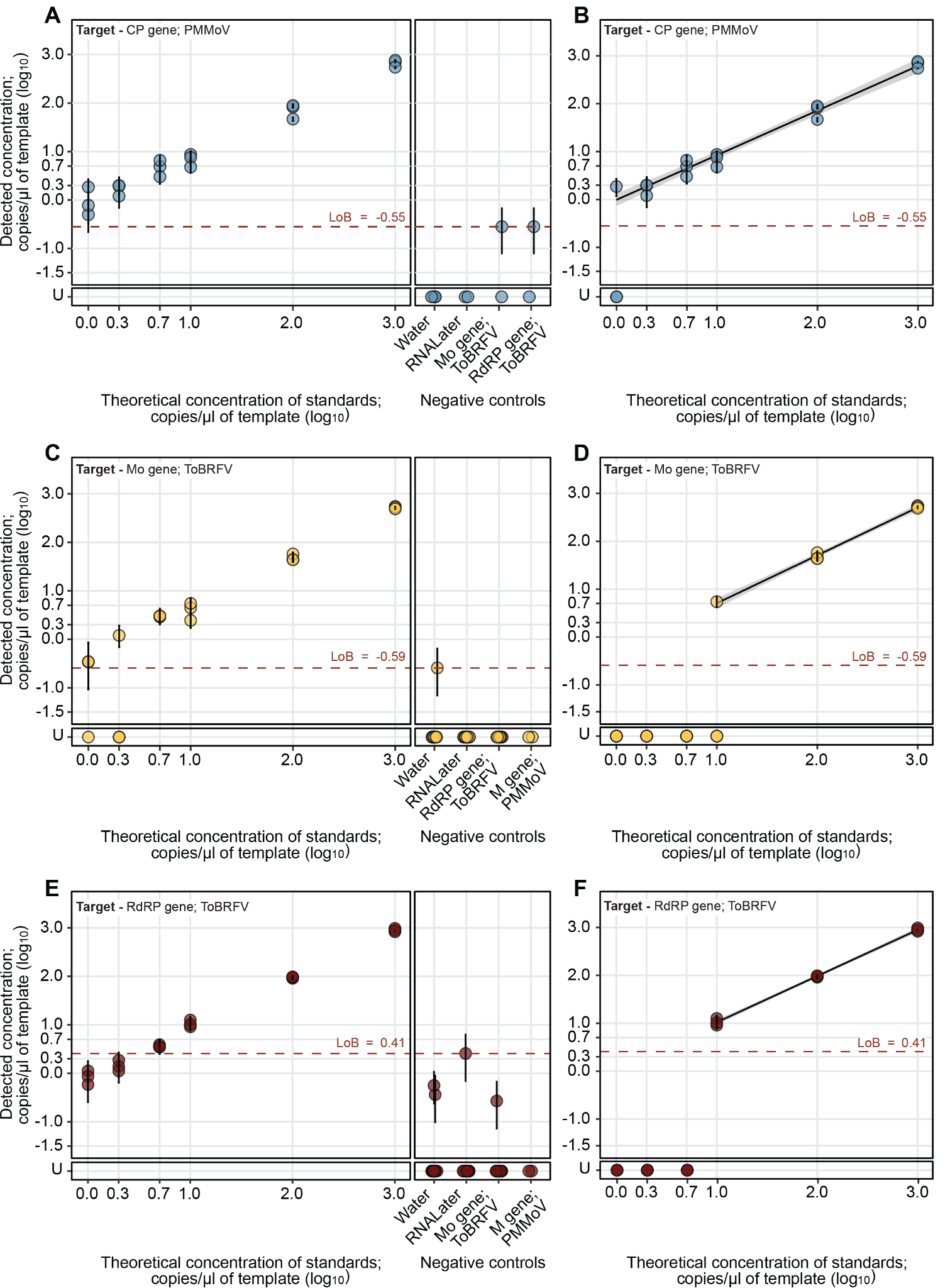


## Figure S4. Analysis of specificity and sensitivity of primer/probes targeting genes in PMMoV and ToBRFV.

The x-axis lists the theoretical concentrations of a synthetic plasmid bearing the PMMoV CP (A and B), ToBRFV Mo (C and D), and RdRP (E and F) genes as standards, paired with matched primer/probes in log_10_ copies/μl, and lists negative samples without the matched target gene. The y-axis lists the gene target concentrations detected using ddRT-PCR in log_10_ copies/ μL of template. Results from three replicates along with their corresponding standard deviation are plotted. The limit of blank (LoB) for each primer/probe combination is marked with a red horizontal line and the corresponding concentrations in log_10_ copies/ μL of template are listed above the line. Panels (A), (C) and (E) represent the raw data from the standard curves. Panels (B), (D) and (F) are plotted after applying the LoB to the raw data, and include the linear regression in black and 95.0% confidence interval in gray. Samples that did not amplify are listed as U for undetermined and not included in the linear regression analysis.

## Figure S5. Prevalence PMMoV and ToBRFV target genes in human stool samples detected by ddRT-PCR.
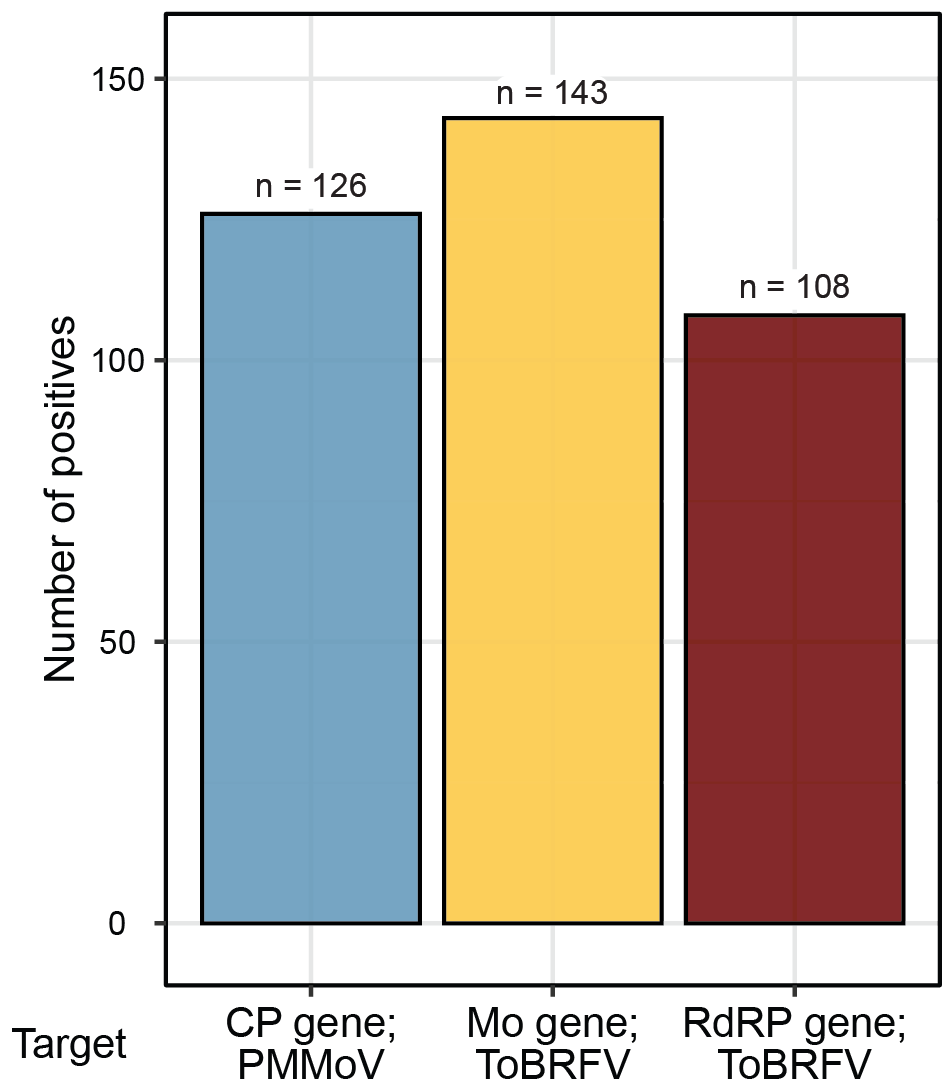


Bar plot summarizing the number of human stool samples containing each of the three target genes. The x-axis marks the target genes and the y-axis lists the number of positive samples. 126/220 (57.3%) samples had the PMMoV CP gene (blue), 143/220 (65.0%) had the ToBRFV Mo (yellow) and 106/220 (48.2%) the RdRP (red) genes respectively.


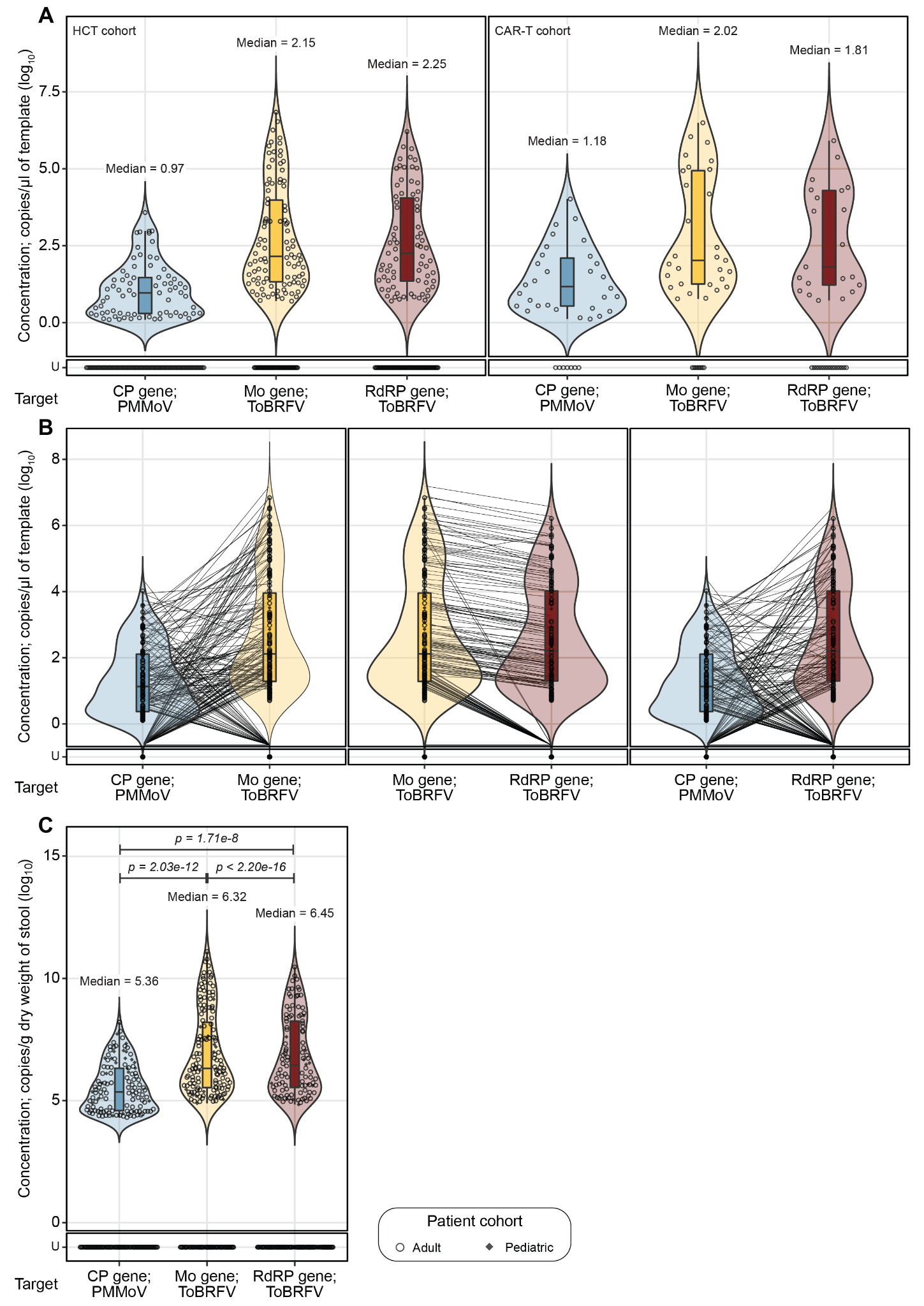


## Figure S6. Concentrations of PMMoV and ToBRFV target genes in human stool samples detected by ddRT-PCR.

Dot plot marking the concentrations of PMMoV CP (blue), ToBRFV Mo (red) and RdRP (yellow) genes, with violin and box plots summarizing their distributions, in RNA extracted from stool samples collected from humans. The x-axis marks the target genes, and the y-axis lists their in log_10_ copies/ μl of template; U stands for “Undetermined” and marks samples with no detectable gene target above LoB. (A) Concentrations of gene targets from samples derived from adults separated by their treatment cohort with those on HCT on the left and CAR-T on the right. In the HCT cohort, the PMMoV CP gene has a median of 0.970 with a standard deviation of 0.886 and IQR of 1.17 log_10_ copies/ μL of template, TOBRFV Mo gene has a median of 2.16 with a standard deviation of 1.68 and IQR of 2.65 log_10_ copies/ μL of template, and ToBRFV RdRP gene has a median of 2.26 with a standard deviation of 1.58 and IQR of 2.69 log_10_ copies/ μL of template. In the CAR-T cohort, the PMMoV CP gene has a median of 1.18 with a standard deviation of 1.07 and IQR of 1.56 log_10_ copies/ μL of template, ToBRFV Mo gene has a median of 2.02 with a standard deviation of 1.89 and IQR of 3.68 log_10_ copies/ μL of template, and ToBRFV RdRP gene has a median of 1.81 with a standard deviation of 1.72 and IQR of 3.07 log_10_ copies/ μL of template. (B) Pairwise analyses of gene target concentrations from the same samples, with adult samples marked by unfilled circles and pediatric samples marked by a filled diamond. Each panel captures analysis from one pair of gene targets, with concentrations derived from the same RNA extract connected by a line. (C) Concentrations of gene targets derived from adult (unfilled circles) and pediatric (filled diamonds) samples expressed in copies/ g dry weight of stool. The PMMoV CP gene has a median of 5.36 with a standard deviation of 0.986 and IQR of 1.74 log_10_ copies/ g dry weight, ToBRFV Mo gene has a median of 6.32 with a standard deviation of 1.69 and IQR of 2.68 log_10_ copies/ g dry weight, and ToBRFV RdRP gene has a median of 6.45 with a standard deviation of 1.57 and IQR of 2.72 log_10_ copies/ g dry weight. *p* values derived from paired Wilcoxon signed-rank tests with continuity correction and excluding samples with undetermined concentration, across all combinations of the three gene targets are listed at the top of the plot.

##
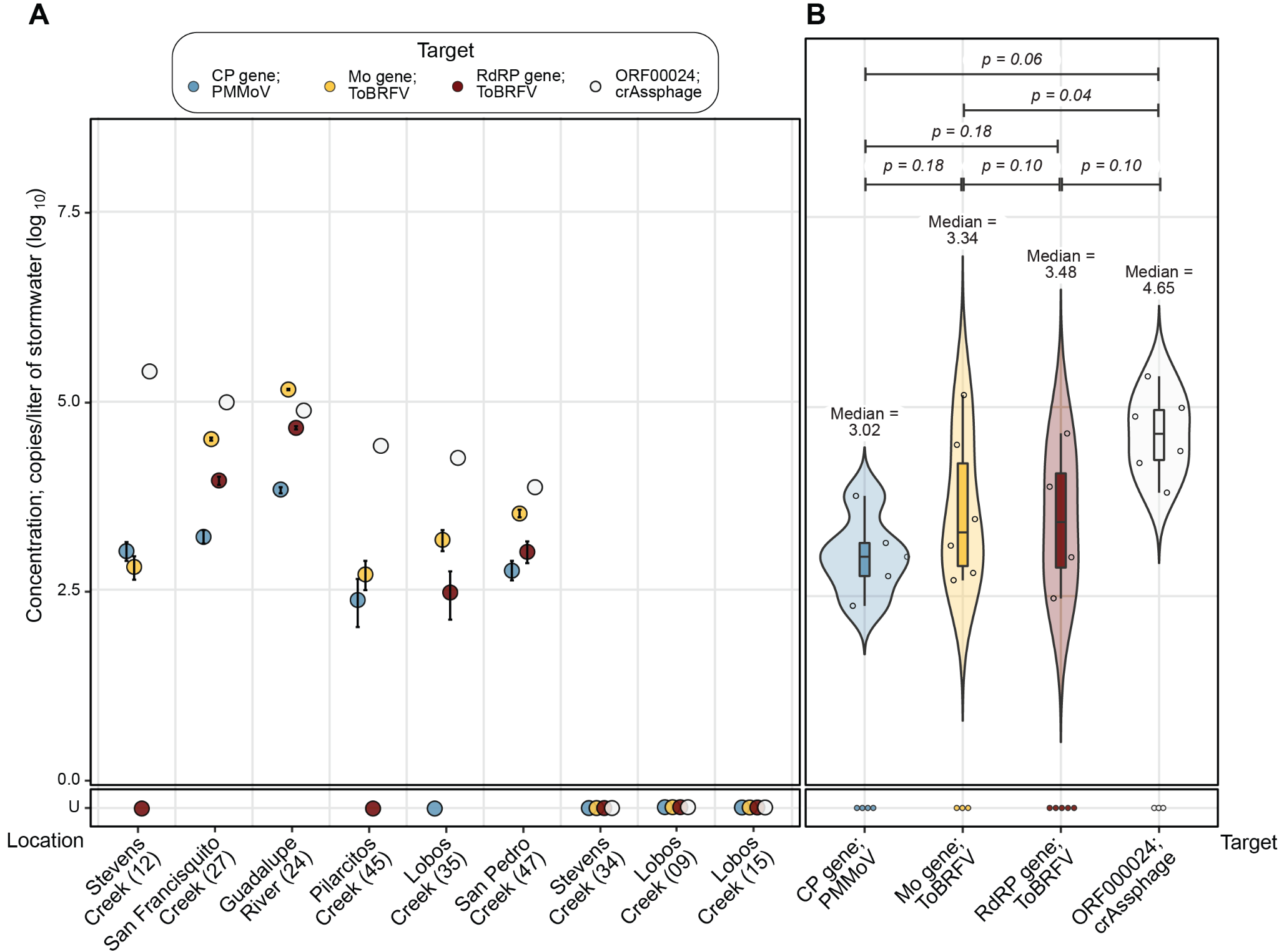
Figure S7. Concentrations of PMMoV, ToBRFV and crAssphage target genes in stormwater samples from across California.

(A) Dot plot marking the concentrations of the PMMoV CP gene (blue), ToBRFV Mo (yellow) and RdRP (red) genes, and crAssphage ORF000024 (white). Data points have error bars marking the associated standard deviation. Data regarding crAssphage concentration is derived from a previous study[(1)](https://paperpile.com/c/xd9hyJ/hhDX) and listed without error bars. The x-axis lists the nine stormwater sources from where samples were acquired followed by sample ID, in decreasing concentration of crAssphage RNA. (B) Dot plot summarizing the concentrations of PMMoV CP (blue), ToBRFV Mo (red), RdRP (yellow) genes, and crAssphage ORF000024 (white) from RNA extracted from stormwater samples, with violin and box plots marking their distributions. The x-axis marks the target genes. The PMMoV CP gene has a median of 3.02 with a standard deviation of 0.54 and IQR of 0.44 log_10_ copies/ liter, ToBRFV Mo gene has a median of 3.34 with a standard deviation of 0.98 and IQR of 1.36 log_10_ copies/ liter, ToBRFV RdRP gene has a median of 3.48 with a standard deviation of 0.97 and IQR of 1.24 log_10_ copies/ liter, and crAssphage ORF000024 has a median of 4.65 with a standard deviation of 0.56 and IQR of 0.66 log_10_ copies/ liter. *p* values derived from paired Wilcoxon signed-rank tests with continuity correction and excluding samples with undetermined concentrations across all combinations of the four gene targets are listed at the top of the plot. The y-axis lists concentrations of the genes in log_10_ copies/ liter of stormwater sample. U stands for “Undetermined” and marks samples with no detectable gene target above LoB.

#
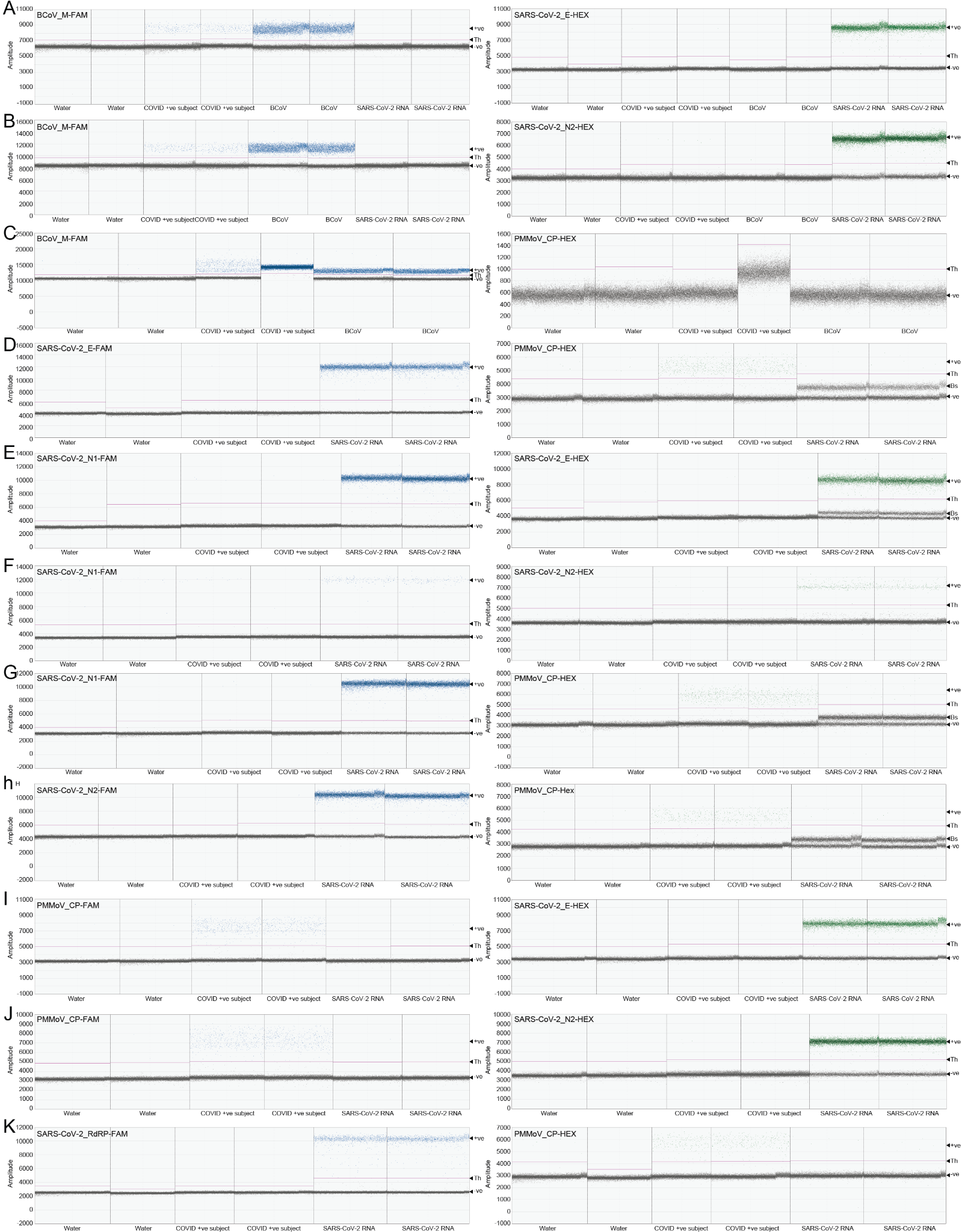


#

## Figure S8. 1D amplitude of ddRT-PCR assays testing compatibility of primer/probes for multiplexed assays.

ddRT-PCR enables the simultaneous detection of two target genes across orthogonal detection channels, one that detects the FAM fluor and the other that detects the HEX fluor. Combinations of the BCoV M gene in channel 1 and SARS-CoV-2 E gene in channel 2 (A), BCoV M gene in channel 1 and SARS-CoV-2 N2 gene in channel 2 (B), BCoV M gene in channel 1 and PMMoV CP gene in channel 2 (C), SARS-CoV-2 E gene in channel 1 and PMMoV CP gene in channel 2 (D), SARS-CoV-2 N1 gene in channel 1 and SARS-CoV-N2 E gene in channel 2 (F), SARS-CoV-2 N1 gene in channel 1 and PMMoV CP gene in channel 2 (G), SARS-CoV-2 N2 gene in channel 1 and PMMoV CP gene in channel 2 (H), PMMoV CP gene in channel 1 and SARS-CoV-2 E gene in channel 2 (I), PMMoV CP gene in channel 1 and SARS-CoV-2 N2 gene in channel 2 (J), SARS-CoV-2 RdRP gene in channel 1 and PMMoV CP gene in channel 2 (K) were evaluated. The x-axis lists relevant sample names as water for the no template control, COVID +ve participant for RNA extracted from a COVID +ve participant admitted to the ICU, SARS-CoV-2 RNA for the synthetic SARS-CoV-2 RNA from ATCC, BCoV for the RNA extracted from attenuated BCoV vaccine. The y-axis lists the amplitude of fluorescence in the respective detection channel. The panels on the left correspond to channel 1 detecting the FAM fluor, and on the right correspond to channel 2 detecting the HEX fluor. In each amplitude plot, where relevant, droplets bearing a positive signal are labeled on the right as +ve, the threshold amplitude is labeled as Th, and droplets bearing a negative signal are labeled as -ve. Raw data are presented in Table S6.

# Supplemental table

## Table S1. Demographic distribution of participants who provided stool for ddRT-PCR

| **Characteristic** | | **Adult (N = 125)** | **Pediatric (N = 4)** |
| --- | --- | --- | --- |
| **Median age (range), years** | | 59.5 (19 - 82) | 6 ( 3 - 16) |
| **Age group - no. (%)** | 10 - 20 | 1 | Not reported** |
|  | 20 - 30 | 12 |  |
|  | 30 - 40 | 17 |  |
|  | 40 - 50 | 11 |  |
|  | 50 - 60 | 25 |  |
|  | 60 - 70 | 45 |  |
|  | 70 - 80 | 12 |  |
|  | 80 - 90 | 1 |  |
|  | Unknown* | 1 |  |
|  | | | |
| **Sex - no. (%)** | Male | 79 | Not reported** |
|  | Female | 45 |  |
|  | Unknown* | 1 |  |
|  | | | |
| **Race - no. (%)** | White | 77 (61.6%) | Not reported** |
|  | Asian | 13 (10.4%) |  |
|  | Black | 6 (4.80%) |  |
|  | Unknown | 25 (20.0%) |  |
|  | Not reported** | 8 (6.40%) |  |
|  | | | |
| **Ethnicity; Hispanic or Latinx - no. (%)** | No | 105 (84.0%) | Not reported** |
|  | Yes | 19 (15.2%) |  |
|  | Unknown | 1 (0.800%) |  |
|  | Not reported** | 4 (3.20%) |  |

*Unknown refers to data that was not provided by participants.

** Not reported refers to data that is aggregated in order to avoid information that can be used to identify participants.

## Table S2. Information on wastewater samples.

| **Sample ID** | **Sample code** | **City** | **State** | **Date of collection (YYYY-MM-DD)** |
| --- | --- | --- | --- | --- |
| Akron | OH-Akn | Akron | OH | 2020-06-10 |
| Boston | MA-Bos | Boston | MA | 2020-06-01 |
| Davis | CA-Dav | Davis | CA | 2021-07-17 |
| Gilroy | CA-Gil | Giloy | CA | 2021-07-17 |
| Hyperion | CA-LA | Los Angeles | CA | 2020-11-17 |
| New York | NY-NYC | New York City | NY | 2020-05-06 |
| North City | CA-SD | San Diego | CA | 2020-11-13 |
| Oceanside | CA-SF | San Francisco | CA | 2021-07-17 |
| Palo Alto | CA-PA | Palo Alto | CA | 2021-07-16 |
| Sacramento | CA-Sac | Sacramento | CA | 2021-07-17 |
| San Jose | CA-SJ | San Jose | CA | 2021-07-16 |
| Silicon Valley | CA-SM | San Mateo | CA | 2021-07-17 |
| Sunnyvale | CA-Sun | Sunnyvale | CA | 2021-07-17 |
| UC Davis | CA-UCD | Davis | CA | 2021-07-08 |
| Wisconsin | WI-Mil | Milwaukee | WI | 2020-09-01 |

##

## Table S3. Information on stormwater samples from California

| **Sample ID** | **Location** | **Date of collection (YYYY-MM-DD)** |
| --- | --- | --- |
| 24 | Guadalupe River | 2018-12-17 |
| 9 | Lobos Creek | 2019-02-21 |
| 15 | Lobos Creek | 2018-10-29 |
| 35 | Lobos Creek | 2019-01-17 |
| 45 | Pilarcitos Creek | 2018-10-10 |
| 27 | San Francisquito Creek | 2018-12-17 |
| 47 | San Pedro Creek | 2018-11-27 |
| 12 | Stevens Creek | 2018-10-10 |
| 34 | Stevens Creek | 2019-01-17 |

##

## Table S4. Sequences of oligonucleotides used as primers and probes

| **Primer** | **Description** | **Sequence (5’ to 3’)** | **Ref** |
| --- | --- | --- | --- |
| PMMoV_CP_F | PMMoV CP gene; forward primer | GAG TGG TTT GAC CTT AAC GTT TGA | [^(2)^](https://paperpile.com/c/xd9hyJ/MqOIQ) |
| PMMoV_CP_R | PMMoV CP gene; reverse primer | TTG TCG GTT GCA ATG CAA GT | [^(2)^](https://paperpile.com/c/xd9hyJ/MqOIQ) |
| BCoV_M_F | BCoV M gene; forward primer | CTG GAA GTT GGT GGA GTT | [^(3)^](https://paperpile.com/c/xd9hyJ/K05YU) |
| BCoV_M_R | BCoV M gene; reverse primer | ATT ATC GGC CTA ACA TAC ATC | [^(3)^](https://paperpile.com/c/xd9hyJ/K05YU) |
| SARS-CoV-2_E_F | E gene forward primer | ACA GGT ACG TTA ATA GTT AAT AGC GT | [^(4)^](https://paperpile.com/c/xd9hyJ/J0rwy) |
| SARS-CoV-2_E_R | E gene reverse primer | ATA TTG CAG CAG TAC GCA CAC A | [^(4)^](https://paperpile.com/c/xd9hyJ/J0rwy) |
| 2019-nCoV_N2-F | N2 gene forward primer | TTA CAA ACA TTG GCC GCA AA | [^(5)^](https://paperpile.com/c/xd9hyJ/WkuEU) |
| 2019-nCoV_N2-R | N2 gene reverse primer | GCG CGA CAT TCC GAA GAA | [^(5)^](https://paperpile.com/c/xd9hyJ/WkuEU) |
|  | | | |
| **Probe** | **Description** | **Sequence (5’ to 3’)** |  |
| PMMoV_CP_P | PMMoV CP gene; probe | CCT ACC GAA GCA AAT G | [^(2)^](https://paperpile.com/c/xd9hyJ/MqOIQ) |
| BCoV_M_P | BCoV M gene; probe | CCT TCA TAT CTA TAC ACA TCA AGT TGT T | [^(3)^](https://paperpile.com/c/xd9hyJ/K05YU) |
| SARS-CoV-2_E_Prb-FAM | E gene probe | ACA CTA GCC ATC CTT ACT GCG CTT CG | [^(4)^](https://paperpile.com/c/xd9hyJ/J0rwy) |
| 2019-nCoV_N2-P | N2 gene probe | ACA ATT TGC CCC CAG CGC TTC AG | [^(5)^](https://paperpile.com/c/xd9hyJ/WkuEU) |

##

## Table S5. Assessment of newly assembled ToBRFV genomes

| **Sample ID** | **Length (bps)** | **Estimated completeness (%)** | **Sample type** |
| --- | --- | --- | --- |
| 20200528WW - Berkeley | 6335 | 99.2 | Wastewater sample |
| 20200609WW - Berkeley | 6301 | 98.7 |  |
| 20200519WW - Oakland | 5976 | 93.6 |  |
| 20200528WW - Oakland | 6341 | 99.3 |  |
| 20200609WW - Oakland | 6350 | 99.4 |  |
| 2020_day1_St - Stanford | 6380 | 99.9 | Stool sample |
| 2020_day10_St - Stanford | 6385 | 100 |  |
| 2020_day93_St - Stanford | 6386 | 100 |  |

##

## Table S6. Summary of template concentrations from all tested multiplexed reactions

| **Channel** | **Target** | **Water** | **RNA from COVID +ve participant** | **Attenuated BCoV vaccine(1:100 dilution)** | **ATCC SARS-CoV-2(10^3 copies/µL)** | **Notes based on Fig. S3** |
| --- | --- | --- | --- | --- | --- | --- |
|  |  | **Concentration (copies/ μL of template)** | | | |  |
| 1 (FAM) | BCoV_M gene | 0.00 | 73.77 | 1840.66 | 0.00 | 1d plot of raw ddRT-PCR amplitude reveals clean separation of signal from background |
| 2 (HEX) | SARS-CoV-2_E gene | 0.00 | 0.30 | 0.00 | 2406.84 |  |
| 1 (FAM) | BCoV_M gene | 0.00 | 76.61 | 1910.56 | 0.14 | 1d plot of raw ddRT-PCR amplitude reveals clean separation of signal from background |
| 2 (HEX) | SARS-CoV-2_N2 gene | 0.00 | 1.30 | 0.14 | 4373.69 |  |
| 1 (FAM) | BCoV_M gene | 0.92 | 14189.80 | 2007.72 | NA | PMMoV_CP-HEX failed |
| 2 (HEX) | PMMoV_CP gene | 0.00 | 0.00 | 0.00 | NA |  |
| 1 (FAM) | SARS-CoV-2_E gene | 0.00 | 0.39 | NA | 2445.72 | 1d plot of raw ddRT-PCR amplitude reveals bleed through of signal from the FAM to the HEX channel |
| 2 (HEX) | PMMoV_CP gene | 2.02 | 171.71 | NA | 1.60 |  |
| 1 (FAM) | SARS-CoV-2_N1 gene | 0.00 | 0.82 | NA | 4055.90 | 1d plot of raw ddRT-PCR amplitude reveals bleed through of signal from the FAM to the HEX channel |
| 2 (HEX) | SARS-CoV-2_E gene | 0.15 | 0.41 | NA | 2454.43 |  |
| 1 (FAM) | SARS-CoV-2_N1 gene | 2.89 | 2.32 | NA | 49.24 | 1d plot of raw ddRT-PCR amplitude reveals that the FAM channel is noisy and leading to false positive RNa concentration in water |
| 2 (HEX) | SARS-CoV-2_N2 gene | 0.00 | 0.58 | NA | 97.56 |  |
| 1 (FAM) | SARS-CoV-2_N1 gene | 0.00 | 0.88 | NA | 4401.05 | 1d plot of raw ddRT-PCR amplitude reveals bleed through of signal from the FAM to the HEX channel |
| 2 (HEX) | PMMoV_CP gene | 1.33 | 169.53 | NA | 2.69 |  |
| 1 (FAM) | SARS-CoV-2_N2 gene | 0.00 | 0.32 | NA | 3620.74 | 1d plot of raw ddRT-PCR amplitude reveals bleed through of signal from the FAM to the HEX channel |
| 2 (HEX) | PMMoV_CP gene | 0.69 | 103.21 | NA | 1.61 |  |
| 1 (FAM) | PMMoV_CP gene | 0.32 | 169.90 | NA | 0.16 | 1d plot of raw ddRT-PCR amplitude reveals clean separation of signal from background |
| 2 (HEX) | SARS-CoV-2_E gene | 0.00 | 0.38 | NA | 2536.65 |  |
| 1 (FAM) | PMMoV_CP gene | 3.30 | 1875.39 | NA | 1.89 | 1d plot of raw ddRT-PCR amplitude reveals clean separation of signal from background |
| 2 (HEX) | SARS-CoV-2_N2 gene | 0.00 | 9.10 | NA | 48239.72 |  |
| 1 (FAM) | SARS-CoV-2_RdRP gene | 0.00 | 0.00 | NA | 490.29 | 1d plot of raw ddRT-PCR amplitude reveals that the HEX channel is noisy and leading to false positive RNa concentration in water |
| 2 (HEX) | PMMoV_CP gene | 0.97 | 105.02 | NA | 1.56 |  |

#

#

# Supplemental notes

## Note S1. Sequencing of total RNA from three stool samples.

RNA extraction was carried out as follows. 300 - 400 mg of each stool sample was added to 2 mL microfuge tubes containing 4 mm zirconium beads. To each sample, 250 μl of Tris-EDTA buffer (pH 7.4), 40 µL of lysozyme (Sigma-Aldrich; Catalog # L3790) at 10 mg/ml, 10 µL Qiagen lytic enzyme solution (Catalog # 158928), and 10 µL metapolyzyme (Sigma-Aldrich; Catalog # MAC4L) at 10 mg/ml were added. Samples were then mixed by vortexing to disrupt the stool matrix, and incubated in a shaker incubator at 37°C at 150 rpm for 15 minutes. Samples were treated with 20 µL of Proteinase K per sample (Qiagen; Catalog # 19157) and incubated for another 15 minutes at 37°C at 150 rpm. To enable mechanical lysis, 1 mL of RLT buffer (Qiagen; Catalog # 79216) with 10 µL of 2-mercaptoethanol was added to each sample, followed by bead beating for 3 minutes and recovery on ice. Debris was removed by centrifugation at 4°C at 21,000 g for 3 minutes. The supernatants were transferred into 5 mL tubes and an equal volume of acidic Phenol/Chloroform (pH 4.5; Invitrogen; Catalog # AM9722) was added to each sample. After vortexing for 3 minutes, the samples were spun down at 12,000 g at 4°C for 10 minutes. The supernatants were transferred to new 5 mL tubes. To purify the RNA from stool-derived contaminants, the supernatants were further purified using the RNA Clean & Concentrator-5 kit (Zymo Research; Catalog # R1013). To each sample, 1 volume of ethanol (95 - 100%) was mixed with the supernatant from the previous step. The samples were transferred to the Zymo-Spin IC Columns in collection tubes and centrifuged. The rest of the purification protocol proceeded according to the kit specifications. The RNA samples were eluted off the columns by adding 30 µL of nuclease-free water directly to the column matrix followed by incubation for 15 minutes at room temperature then centrifugation into collection microfuge tubes. All samples were stored at -80°C for up to 14 days until they were processed for RNA sequencing. Total RNA extracted from stool samples was subjected to rRNA depletion using a pre-commercial version of the RiboZero Plus Microbiome kit (Illumina). Following depletion, the samples were converted into libraries using the Illumina RNA Prep for Enrichment kit. To obtain shotgun metatranscriptomic information from the rRNA-depleted samples, the pre-enriched (Total RNAseq) libraries were sequenced on a NextSeq 550.

##

## Note S2. Identifying primers/probes that are compatible in multiplexed ddRT-PCR assays

The QX200 ddPCR droplet reader enables the simultaneous detection or multiplexing of nucleic acids across two fluorescence channels where - channel 1 measures wavelength corresponding carboxyfluorescein (FAM) and channel 2, hexachlorofluorescein (HEX). Since we planned to assay for four target genes, two corresponding to the SARS-CoV-2 genomic RNA, and one each for the PMMoV and BCoV genomic RNA, we sought to maximize our throughput and save RNA extracted from precious clinical samples by multiplexing our reactions. However, this required us to evaluate the performance of primer/probe combinations targeting relevant genes. Notably, we want to pick combinations of primer/probes bearing orthogonal fluorophores such that they retain high signal-to-noise separation in their respective channels of detection while also avoiding interference of signal across channels. This is evaluated based on three observations in the 1-D amplitude plots from ddRT-PCR data:

1. Rain: In an ideal ddRT-PCR reaction, the amplitude of the droplets that are positive will cluster distinctly at a higher value than the amplitude of the droplets that are negative. However, when the reaction is not efficient, there ends up being a number of droplets that have an intermediate amplitude between the positive and negative values. These are referred to as “rain”. We want to pick primer/probes that have none to minimal rain.
2. Separation in amplitude between signal and noise: As previously mentioned, in ddRT-PCR, droplets that bear a positive signal will have a higher mean amplitude compared to the negative droplets. We want to pick primer/probes that had maximal differences between the mean positive and negative amplitude. This allows us to comfortably set the threshold between the positive and negative amplitudes.
3. Signal bleed through across channels: Theoretically, the FAM and HEX fluorescence signals are meant to be orthogonal, meaning a positive signal in one channel should not affect a read-out in the other. This is crucial to accurately quantify two different gene targets in a single reaction. However, some primer/probes lead to bleeding through of signal across channels. This means that where we should see droplets form clusters around two amplitudes for the positives and negatives respectively, we instead witness a third cluster representing signal from the orthogonal channel. The presence of bleed-through in the signal will interfere with the accurate quantification of target genes.

The United States Centers for Disease Control and Prevention (CDC) [(6)](https://paperpile.com/c/xd9hyJ/86wa6) and the German Centre for Infection Research (DZIF) [(4)](https://paperpile.com/c/xd9hyJ/J0rwy) suggest four gene targets for PCR-based detection of SARS-CoV-2. These are the genes encoding the Envelope protein (E), Nucleocapsid proteins (N1, N2), and RNA-dependent RNA polymerase protein (RdRP). Previous literature has optimized primer/probes targeting the gene for the coat protein (CP) in PMMoV [(2)](https://paperpile.com/c/xd9hyJ/MqOIQ) and transmembrane protein (M) in BCoV [(3)](https://paperpile.com/c/xd9hyJ/K05YU). In a bid to find the best combination of primer/probes, we acquired oligonucleotides targeting the E, N1, N2, RdRP, CP and M genes tagged with FAM, and the E, N2 and CP genes tagged with HEX (Table S4). The repertoire of HEX labeled primer/probes tested herein was limited by the availability of reagents.

The raw 1-D amplitudes from these 11 combinations of primers and probes were analyzed to identify multiplexed reactions that did not feature rain and signal bleed through across channels (Fig. S8) and presented the best separation of signal from noise using appropriate control samples. Water is used as a universal no template control and synthetic SARS-CoV-2 viral RNA from ATCC is used as a positive control for SARS-CoV-2 target genes. Viral RNA extracted from 1) stool from a COVID-19 +ve participant who was admitted to the ICU is used as a positive control for SARS-CoV-2 and PMMoV viral RNAs, and 2) attenuated BCoV vaccine is used as a positive control for BCoV. These samples also serve as negative controls for the complementary primers and probes. Through this analysis, we identified that multiplexing the detection of the SARS-CoV-2 E gene and the PMMoV CP gene, and the SARS-CoV-2 N2 gene and the BCoV M gene in two independent multiplexed reactions using the FAM and HEX fluors respectively performed the best (Fig. S8, Table S6).

# References

1. [Graham KE, Anderson CE, Boehm AB. 2021. Viral pathogens in urban stormwater runoff: Occurrence and removal via vegetated biochar-amended biofilters. Water Res 207:117829.](http://paperpile.com/b/xd9hyJ/hhDX)

2. [Haramoto E, Kitajima M, Kishida N, Konno Y, Katayama H, Asami M, Akiba M. 2013. Occurrence of pepper mild mottle virus in drinking water sources in Japan. Appl Environ Microbiol 79:7413–7418.](http://paperpile.com/b/xd9hyJ/MqOIQ)

3. [Decaro N, Elia G, Campolo M, Desario C, Mari V, Radogna A, Colaianni ML, Cirone F, Tempesta M, Buonavoglia C. 2008. Detection of bovine coronavirus using a TaqMan-based real-time RT-PCR assay. J Virol Methods 151:167–171.](http://paperpile.com/b/xd9hyJ/K05YU)

4. [Corman VM, Landt O, Kaiser M, Molenkamp R, Meijer A, Chu DK, Bleicker T, Brünink S, Schneider J, Schmidt ML, Mulders DG, Haagmans BL, van der Veer B, van den Brink S, Wijsman L, Goderski G, Romette J-L, Ellis J, Zambon M, Peiris M, Goossens H, Reusken C, Koopmans MP, Drosten C. 2020. Detection of 2019 novel coronavirus (2019-nCoV) by real-time RT-PCR. Euro Surveill 25.](http://paperpile.com/b/xd9hyJ/J0rwy)

5. [2020. CDC 2019-Novel Coronavirus (2019-nCoV) Real-Time RT-PCR Diagnostic Panel. CDC-006-00019, Revision: 06. Division of Viral Diseases, Centers for Disease Control and Prevention.](http://paperpile.com/b/xd9hyJ/WkuEU)

6. [Lu X, Wang L, Sakthivel SK, Whitaker B, Murray J, Kamili S, Lynch B, Malapati L, Burke SA, Harcourt J, Tamin A, Thornburg NJ, Villanueva JM, Lindstrom S. 2020. US CDC Real-Time Reverse Transcription PCR Panel for Detection of Severe Acute Respiratory Syndrome Coronavirus 2. Emerg Infect Dis 26.](http://paperpile.com/b/xd9hyJ/86wa6)
